# Cellular, Synaptic and Network Effects of Acetylcholine in the Neocortex

**DOI:** 10.3389/fncir.2019.00024

**Published:** 2019-04-12

**Authors:** Cristina Colangelo, Polina Shichkova, Daniel Keller, Henry Markram, Srikanth Ramaswamy

**Affiliations:** Blue Brain Project, Ecole Polytechnique Fédérale de Lausanne (EPFL), Geneva, Switzerland

**Keywords:** neuromodulation, acetylcholine, Ach receptors, neocortex, cellular excitability, synaptic transmission, network activity

## Abstract

The neocortex is densely innervated by basal forebrain (BF) cholinergic neurons. Long-range axons of cholinergic neurons regulate higher-order cognitive function and dysfunction in the neocortex by releasing acetylcholine (ACh). ACh release dynamically reconfigures neocortical microcircuitry through differential spatiotemporal actions on cell-types and their synaptic connections. At the cellular level, ACh release controls neuronal excitability and firing rate, by hyperpolarizing or depolarizing target neurons. At the synaptic level, ACh impacts transmission dynamics not only by altering the presynaptic probability of release, but also the magnitude of the postsynaptic response. Despite the crucial role of ACh release in physiology and pathophysiology, a comprehensive understanding of the way it regulates the activity of diverse neocortical cell-types and synaptic connections has remained elusive. This review aims to summarize the state-of-the-art anatomical and physiological data to develop a functional map of the cellular, synaptic and microcircuit effects of ACh in the neocortex of rodents and non-human primates, and to serve as a quantitative reference for those intending to build data-driven computational models on the role of ACh in governing brain states.

## Introduction

The cholinergic system is one of the most well-studied neuromodulatory systems, and perhaps phylogenetically the oldest. Acetylcholine (ACh) is found in both vertebrates and invertebrates and together with adrenaline and noradrenaline (NA), it acts as one of the main effectors of the autonomic nervous system. In the central nervous system (CNS), ACh impacts cellular and synaptic physiology and may switch network dynamics resulting in behavioral transitions such as from sleep to wakefulness, distraction to attention, and learning and recall (Hasselmo and Sarter, 2011; Lee and Dan, 2012).

Cholinergic effects have been studied for more than a century. In 1869, Schmiedeberg and Koppe (1869) demonstrated how extracts of a common mushroom, *Amanita muscaria*, could slow, and at a higher concentration arrest the beat of the frog heart. They purified the extract and named it muscarine. This substance, when applied to the brain and spinal cord was able to produce flaccidity and weaken the peripheral reflexes. However, the pharmacology of the nitrite ester of choline was different in that it had considerable nicotinic activity (nicotine is the major alkaloid of tobacco, first isolated by Posselt and Reiman from *Nicotiniana tabacum* leaves in 1828; Koukouli et al., 2017). In 1921 experimental proof was obtained for ACh’s role as a chemical transmitter at the cardiac vagal endings. The active substance was initially named “vagusstoff” by Otto Loewi in 1921 (Loewi, 1924). Sir Henry Dale further described that muscarinic responses were antagonized by atropine, whereas the nicotine actions were antagonized by curare (Dale, 1914).

It has long been known that ACh is also present at the level of the CNS, however, it was not until 1953 that evidence of the release of ACh in the brain was provided (Eccles et al., 1953). Prior to this discovery, it was known that anti-cholinergic drugs could influence learning and memory—pharmacological activation of muscarinic ACh receptors (mAChRs) was known to produce delirium symptoms, while receptor blockade generates severe anterograde amnesia. Moreover, the dementia of Alzheimer’s and Parkinson’s diseases has been associated with the loss of cortical cholinergic innervation (Little et al., 1998; Giacobini, 2003; Sabri et al., 2008; Hasselmo and Sarter, 2011), and chronic administration of nicotine reverses hypofrontality in animal models of addiction and schizophrenia (Koukouli et al., 2017).

Classical notions sustain the view that the central cholinergic system works by a diffuse release of ACh across the cortex, activating its receptors globally and producing slow responses. While this view might be applicable to long-lasting behavioral phenomena, such as cortical arousal, it does not explain the modulation of processes that happen on a much faster scale, such as sensory gating, or plasticity (Muñoz and Rudy, 2014). ACh release in the neocortex originates from neurons distributed within the basal forebrain (BF) nuclei, including the medial septum, the vertical and horizontal diagonal band of Broca, the substantia innominata, and the nucleus basalis of Meynert (NBM). Release occurs through topographical projections, and all the projections arise from six groups of choline acetyl-transferase (ChAT)-positive neurons in the BF (Ch1–Ch4) and brainstem (Ch5–Ch6; Wevers, 2011). The innervation sparsely reaches all cortical layers, but layer 5 is more heavily innervated, particularly in the motor and sensory areas; cholinergic pathways often provide *en passant* innervation (Dani and Bertrand, 2007) to the neocortex. Additionally, ACh-releasing cells are found in cortical layer 2/3. These cells exhibit a bipolar morphology, stain positive for calretinin (CR) and vasoactive intestinal peptide (VIP), and are GABAergic (von Engelhardt et al., 2007; Granger et al., 2018).

The function of a neuromodulatory system is largely defined by the anatomy of its projections. Projections from the BF selectively control cortical activity and target neocortical regions more specifically than previously assumed (Hasselmo and Sarter, 2011; Muñoz and Rudy, 2014; Obermayer et al., 2017). Recent evidence suggests that a roughly topographical organizational scheme exists in the rostro-caudal sequence of neurons of the BF (Zaborszky et al., 2015) and that specific BF nuclei innervate specific cortical areas, as opposed to what happens with noradrenergic fibers originating from the locus coeruleus (Chaves-Coira et al., 2016; Kim et al., 2016). Cholinergic fibers can take one of four different routes to cortical structures: the septal pathway (which projects mainly to the hippocampal cortex) the medial pathway, the lateral pathway, or the internal capsule projection (which preferentially project to the neocortex; Poorthuis et al., 2014). Cholinergic terminals that reach the neocortex, mainly *via* layer 1 or layer 6 (Obermayer et al., 2017), can either exert a spread out control of cortical activity and regulate processes such as the transition from sleep to wakefulness and arousal, or contact a restricted number of cortical elements and have cell-type specific effects; here contextual cholinergic signals act in concert with local processing of sensory inputs in order to guide behavior.

The aim of this review is to bring together current knowledge of cholinergic modulation in the neocortex and to identify the gaps to propose future directions to advance the field of neuromodulation. Here, we summarize existing literature on ACh release in the neocortex of rodents and non-human primates, specifically focusing on how ACh-induced effects on the diversity of cell-types and synapses shape the emergence of network states and review theories that bridge the modulation of local circuit properties and the consequent reconfiguration of cortical states. Data-driven computational models allow predictions on the potential role of ACh in reconfiguring neocortical states (Ramaswamy et al., 2018). Therefore, this review reconciles the minimal, although sparse, datasets required to build a multi-scale computational model of the neocortical cholinergic system.

## Volume vs. Synaptic Transmission

A major factor that determines the spatiotemporal precision of ACh action is the transmission mode at cholinergic terminals. Cholinergic cortical signaling has historically been considered a slow and diffuse process, which was established upon examination of the functional organization of cholinergic projections and was mainly based on reports indicating a nearly complete absence of classical synapses at the level of cholinergic terminals (Muñoz and Rudy, 2014). Before optogenetic techniques were available, cholinergic pathways could not be activated in a selective manner, and thus evidence of the existence of fast cholinergic synaptic transmission was lacking, although some proof of fast nicotinic responses was already available from hippocampal recordings (Kalmbach et al., 2012; Obermayer et al., 2017).

In the cerebral cortex, cholinergic fibers are distributed in an intricate network with a characteristic laminar pattern. They have a higher density in the deeper layers. Cholinergic innervation reflects the classic organizational scheme of information processing systems (Kennedy and Bullier, 1985), with a higher number of projections being present in higher-order areas. Presumed cholinergic release sites have been ultra-structurally inspected and the subtle presence of synapse-like contacts has indeed been revealed; however, a relatively large number of these small varicosities, which are often associated with accumulated synaptic vesicles, do not seem to effectively establish synaptic contact with neighboring neurons, or exhibit only a few morphologically identifiable synapses Furthermore, the scarceness of astrocytic processes in the immediate vicinity of ChAT-immuno-reactive axons (when compared to glutamatergic terminals) may also allow greater diffusion of ACh within the extracellular space (Aoki and Kabak, 1992). Thus, relatively low concentrations of ACh will reach locations that are distant from the release site. This produces volume transmission or bulk release: neuromodulators slowly diffuse in a wide cortical area and bind to a large pool of extra-synaptic receptors (Dani and Bertrand, 2007).

Many studies (Umbriaco et al., 1994; Descarries and Mechawar, 2000; Sarter et al., 2009; Yamasaki et al., 2010) conducted in the neocortex have suggested that ACh acts preferentially non-synaptically; however, central cholinergic synapses had already been observed in the early ‘90s. Actual synapses were found on cholinergic varicosities in the cingulate cortex of the rat (Umbriaco et al., 1994), and in macaque more than 40 percent of cholinergic varicosities contained synaptic specializations (Mrzijak et al., 1995). Later, Turrini et al. (2001) provide definitive evidence that suggests that synaptic mechanisms of cholinergic transmission not only exist but prevail in the rat neocortex. Ultrastructural observations that most (66%) cholinergic boutons—as revealed by IR assays for the specific cholinergic marker, vesicular ACh transporter (vAChT)—establish classical synapses in layer 5 of the rat parietal cortex. By applying an improved fixation protocol and by using an antibody for vAChT, Turrini et al. (2001) demonstrated that cholinergic boutons predominantly established symmetric synapses on layer 5 dendritic shafts. The authors also found that immuno-stained varicosities occasionally established asymmetric contacts, but always on dendritic spines.

Another study probed the molecular-anatomical relationship between detectable cholinergic varicosities and the most abundant receptor subtype in the cortex—the muscarinic receptor subtype M1 (Yamasaki et al., 2010). This study established that in the mouse neocortex M1 can be found almost exclusively on the extra-synaptic membrane of pyramidal cells (PCs). Here, they observed that M1 distribution is far denser than the putative cholinergic release sites and that it does not show any apposition pattern to the varicosities, nor to the cytomatrix active zone proteins that are normally found at glutamatergic terminals. Hence, M1’s function in cortical PCs may be to sense ambient ACh released from cholinergic terminals at variable distances, and the main modality through which it is recruited is likely to be volume transmission. These approaches not only contribute to building a more refined knowledge of the subcellular localization of receptor subtypes but also provide a method to qualitatively discriminate between two major modes of transmission. Because of a substantial difference in the distribution pattern of cholinergic receptors across species, it is very likely that experiments performed in different species will yield conflicting results. For instance, even though a low incidence of classical synapses was reported for the rodent brain, a much higher proportion of cholinergic synapses was found in primates (Smiley et al., 1997). In the human cerebral cortex, the same authors found that up to 67% of all cholinergic varicosities established synaptic contacts, suggesting that ACh signaling in humans is mostly mediated by point-to-point synaptic transmission; this mechanism appears to prevail in the primate brain, but whether the same can be said for rodents is still a matter of open debate.

Cholinergic innervation from the BF is more specific than previously considered; ACh can control cortical activity on a fine spatial scale as well. Indeed, these findings agree with the evidence of ACh signaling occurring through direct fast point-to-point synaptic transmission brought about by the application of optogenetic tools (Kalmbach et al., 2012). Overall, it is not completely clear yet whether one mode of cholinergic transmission prevails over the other. Instead, a growing body of evidence suggests that volume and synaptic transmission may be complementary mechanisms by which ACh modulates cortical function (Sarter et al., 2009). While bulk release is thought to cause a more tonic change in extracellular ACh concentration, in the scale of seconds and minutes, and is mainly mediated by activation of extra-synaptic receptors, ACh release occurring at junctional sites would have a more circumscribed influence, with the modulation of circuit activity being restricted to the contacted cortical elements and to a much more delimited spatiotemporal scale (Muñoz and Rudy, 2014). Taken together, evidence shows that ACh modulates microcircuit activity with different modalities, ranging from synaptic release to volume transmission, and exerts its effects by modifying membrane excitability or synaptic activity.

Instead of trying to proclaim one modality over the other, future research should address the issue of whether they can occur simultaneously and have a differential impact on the temporal aspects of the response. Traditional bath application of agonists results in broad spatial and temporal activation that might not reflect the accuracy of endogenous ACh release (Urban-Ciecko et al., 2018). It is thus of crucial importance to determine whether the different ways in which cholinergic agonists are experimentally applied reflect different transmission modalities, and how faithfully stimulation protocols replicate physiological conditions. In the future, ACh application should be standardized according to precisely obtained dose-response and sensitization kinetics curves, and ascending concentrations should be used in order to detect eventual dose-dependent responses. Furthermore, it would be of outstanding interest to better understand how ACh release obtained by optogenetic stimulation of cholinergic afferents compares against bath application of cholinergic agonists. In a recent study, optogenetic recruitment of cholinergic fibers was performed in parallel with 1 mM ACh bath-application to detect changes in Martinotti cells (MCs) activity: the two techniques yielded very similar results (Obermayer et al., 2018). Perhaps the high concentration of ACh used in this case is comparable with a more physiological activation of the cholinergic system. Further clarification is required on the matter, and future studies should, therefore, consider this issue and design their experiments accordingly.

Cholinergic projections are likely to be arranged according to a modular pattern, with isolated bands of neighboring ChAT^+^ cells in the BF having defined cortical targets that are, in turn, functionally associated. When retrograde dyes are injected in distant cortical areas, labeled regions of cholinergic cells in the BF still largely overlap, even though the innervated cortical space is quite restricted (Muñoz and Rudy, 2014). Furthermore, Zaborszky et al. (2015) assert that the degree of overlap of labeled neuronal locations within the BF is positively correlated to the connection strength between the different injected cortical regions. Such an organization could induce widespread modulation even when the system is only focally activated (Muñoz and Rudy, 2014). Nevertheless, the response to neuromodulatory inputs is determined by the interplay of multiple factors, such as post-synaptic target, receptor type and subunit composition, subcellular localization of the receptors and their sensitivity. This way, a diffusely-organized projection system can fine-tune microcircuit activity. The cholinergic projection system should be viewed as a highly dynamic structure, able to propagate inputs either selectively or diffusely, switching from one modality to another, depending on the needs.

The next section aims to unravel the contribution of each subtype of cholinergic receptor to microcircuit modulation and attempts to determine the physiological relevance of their compartmentalized distribution and differential activation.

## Cholinergic Receptors

Even though the differential pharmacological effects had already been characterized, it was not until the early 1950s that the idea of “receptors” as the binding site for ACh was firmly established by Eccles et al. (1953). Cholinergic receptors are composed of two classes of transmembrane macromolecular complexes, the muscarinic and the nicotinic receptor families, each of which is further divided into subclasses. The occurrence of many ACh receptor subtypes and their differential dendritic, somatic, axonal, and synaptic localization contribute to the varied roles that these receptors play in the CNS. Cholinergic receptors have been found on axons originating from thalamic, cortical or basalo-cortical fibers as well as on cortical pyramidal excitatory neurons and inhibitory GABAergic interneurons (Groleau et al., 2015). The precise layer-wise distribution of cholinergic terminals, the identification of cell-types that actually express cholinergic receptors, and the subcellular localization of these receptors are described in the following sections.

## Muscarinic Receptors

Cholinergic synapses throughout the CNS are composed of muscarinic receptors (mAChRs), which can be further differentiated into subtypes that are encoded by a single gene (Venter et al., 1988; Van der Zee and Luiten, 1999). Five genetically defined and pharmacologically characterized (M1 to M5) mAChR subtypes have been identified in the CNS with high levels of expression in subcortical structures and the cerebral cortex (Wevers, 2011). Immunocytochemical approaches have identified different levels of expression of mAChRs throughout the cerebral cortex. These studies have detected moderate levels of mAChRs in the frontal cortex, parietal cortex, temporal cortex, entorhinal cortex, occipital cortex, insular and cingulate cortex, with the highest values for the temporal and occipital cortex. M1 receptors are the most abundantly expressed among all subtypes of mAChRs (Wevers, 2011). The density of cholinergic terminals in the rat neocortex differs between the six layers and depends on the cortical region studied (Eckenstein et al., 1988; Lysakowski et al., 1989). The pattern of cellular staining for mAChRs in the neocortex is characterized by a clear laminar distribution: in most of the cortical mantle, especially in neocortical areas, predominantly layer 5 PCs (L5PCs) show strong immunoreactivity across mammals such as the mouse, golden hamster, rat, cat, and human (Van der Zee and Luiten, 1999).

The density of each mAChR subtype differs throughout the brain with M1 being the most abundantly expressed and M5 the least (Alger et al., 2014). In the hippocampus and neocortex, M1 is present at high levels, M3 is moderately represented (though generally low elsewhere) and M4 is present in high density, as almost anywhere else in the brain, even though its concentration is considerably lower than M1. M2 instead, is found at very low densities, and this class of receptors seems to be distributed according to a precise pattern. M2 receptors frequently reside on presynaptic axonal terminals, whereas M1 receptors are often located on somato-dendritic regions of neurons. The M5 subtype is believed to play an important role in cortical perfusion, and it is mainly expressed on endothelial cells of the cerebral vascular system (Elhusseiny and Hamel, 2000; Gericke et al., 2011) even though recent evidence suggests that the M3 subtype is also involved in this kind of process (Zuccolo et al., 2017). In the rodent visual cortex, the subtypes M1 and M2 predominate, while in primates the subtypes M1, M2 and M4 prevail. Besides a few regional variations, highest labeling densities have been observed in the superficial layers of most cortical areas for both M1 and M2 (Wevers, 2011).

Most cholinergic receptors are metabotropic and mediate slow responses, which are typically associated with volume transmission. In the neonatal and adult cortices of rodents and primates, M1–M5 subtypes of mAChRs occur in both pre-synaptic and post-synaptic positions (Mrzljak et al., 1993; Groleau et al., 2015). All mAChRs are transmembrane macromolecular complexes that are coupled to membrane-embedded G-proteins of different kinds; g-proteins act as intracellular effectors and initiate signaling cascades that ultimately have an effect on intracellular processes, leading to the opening or closing of some ion channel, or to the production of long-term modifications of genetic activity and protein expression. Different mAChRs are coupled to specific G-proteins. The pre-synaptic mAChRs M2 and M4 preferentially couple to G_i_ and G_o_ proteins that generally have inhibitory effects on voltage-activated calcium channels or extend the opening of potassium channels. The resulting decrease in c-AMP signaling suppresses neurotransmitter release (Groleau et al., 2015). M1, M3 and M5 subtypes are preferentially coupled to G_q_ and G_11_ proteins and are mainly located post-synaptically. Their activation seems to trigger membrane depolarization and increases the input-resistance of the cell membrane. M1-like (M1-M3-M5) receptors are known to potentiate NMDA currents and also influence and modulate voltage-dependent calcium currents, mostly by upregulating phospholipase C (PLC) signaling and inositol triphosphate (IP_3_) turnover. One major effect that can be attributed to M1-type receptors is the inhibition of potassium currents, including the I_m_ and the I_AHP_ (both medium and slow rate). However, M1-type receptors can also potentiate cationic currents like the I_h_ and the TRP currents, and the I_cat_ (Teles-Grilo Ruivo and Mellor, 2013). For a more detailed description of the effects of ACh on various currents and their associated intracellular signaling pathways, we direct the reader to the section “Subcellular Nicotinic and Muscarinic Pathways” of this review.

## Pre-synaptic Localization

What anatomical and functional evidence exists on the distribution of mAChRs in the neocortex? Muscarinic cholinergic activity influences sensory processing by facilitating or depressing neuronal responses to specific stimuli, and by modulating connections strength and neural synchronization: this results in the fine-tuning of cellular and network properties during developmental processes, the execution of attention tasks and perceptual learning (Groleau et al., 2015). These effects can largely be attributed to M1 and M2 subtypes, which appear to be highly prevalent in the neocortex. The presence of M1 and M2 mAChRs on PC somata and apical dendrites in non-human primates is well established, but M2 receptors are also found on excitatory and inhibitory axons in the primate neocortex (Mrzljak et al., 1993). Disney et al. (2006) report that M1 and M2 receptor labeling can be observed, but is quite weak in axons and terminals in the macaque visual cortex, whereas mAChRs are mostly expressed at the level of the soma of GABAergic neurons and in the dendritic compartments of glutamatergic cells.

Among the presynaptic receptors in the rodent and human visual cortex, M2 is very abundant while M4 is less prevalent (Groleau et al., 2015). M2 and M4 are mostly found at the presynaptic terminals; activation of these receptor subtypes causes membrane hyperpolarization and conveys a self-inhibitory signal. Thus, extracellular levels of ACh are regulated by means of negative feedback. In the rat’s primary visual cortex (V1) M2 is mainly found at the level of cholinergic terminals in layer 4 and layer 5. Being the main inhibitory auto-receptor, it contributes to the suppression of presynaptic ACh release (Mrzljak et al., 1993).

It is not yet clear whether the presence of M2-like subtypes at the level of the presynaptic terminal is a distinctive feature of cholinergic axons innervating the neocortex. Conflicting results emerge when looking at rodent studies, while experiments done on non-human primates and cats corroborate M2 receptors as the main auto-receptors localized on BF cholinergic axons. Subsequent research should, therefore, address this issue and determine the extent to which presynaptic M2-like receptors account for negative feedback *via* auto-inhibition, since this type of self-regulatory process is crucial for the fine-tuning of the response. Moreover, given that BF fibers originating from distinct neuron clusters differentially innervate separate cortical areas (Zaborszky et al., 2015; Chaves-Coira et al., 2016; Kim et al., 2016), discrepancies should be expected when assessing receptor subtype distributions across neocortical regions. Estimation of the physiological presynaptic distribution profile of inhibitory auto-receptors in the rodent sensory cortex is of key importance to understanding the system’s self-calibrating features. A systematic anatomical profiling of receptor expression should be performed in the rodent models, and quantitative comparisons should be made across sensory areas.

## Post-synaptic Localization

Neocortical PCs and inhibitory interneurons are strongly innervated by cholinergic axons, with L5PCs being the most densely innervated cells; however, numerous immuno-reactive interneurons can be found in all layers, but most frequently in layer 2/3 and layer 5. Here, the mAChR positive interneurons are intermingled with labeled PCs, but in general, the immunostaining of interneurons is less dense than that of the PCs (Van der Zee and Luiten, 1999). While mAChRs are more easily found in the dendritic compartments of PCs, their expression profile throughout the diversity of inhibitory interneurons is quite homogeneous, as these receptors are detected in proximity of the somatic compartment (Disney et al., 2006). mAChRs are expressed by different types of interneurons. In macaque, M2 receptors are found in 31% of PV neurons, 23% of CB neurons, and 25% of CR neurons. 87% of PV^+^ neurons, 60% of CB^+^ neurons and 40% of CR^+^ neurons however, express M1-type mAChRs. The M1 subtype is found across the cortical mantle on the cell bodies and dendrites of post-synaptic PCs, and it appears to be present mainly in layers 2/3 and 6, but it can be found across all cortical layers. In macaque V1, M1 is mostly expressed on GABAergic interneurons, but it is also found on cortico-cortical fibers (Mrzljak et al., 1993; Groleau et al., 2015). M1 immuno-reactivity is also observable in interneurons of the rat neocortex (Levey et al., 1991), although other studies have pointed to a low expression of M1 in primary sensory cortices of rats, such as S1 and V1. Some found M1 expression on PV^+^ neurons to be low or even undetectable in mice neocortex (Yamasaki et al., 2010). The significant difference in expression between rodents and primates could be explained by the fact that M1 receptors are much more associated to the extra-synaptic membrane compartments and are usually activated by volume transmission. Given that the BF cholinergic projection system is scaled-up in primates relative to rodents, there could be a more widespread distribution of M1 receptors throughout cortical interneurons. M1 immuno-reactivity is also detected at the synaptic level, in both inhibitory and excitatory synapses across cortical layers, but more frequently on asymmetric synapses, and here, preferentially on dendritic spines, as opposed to symmetric synapses where M1 is found mostly on dendritic shafts (Mrzljak et al., 1993). This preferential distribution perspective is challenged though, by experimental evidence that cholinergic boutons form synapses mainly with dendritic shafts, much fewer with dendritic spines and only occasionally on neuronal somata (Beaulieu and Somogyi, 1991; Mrzljak et al., 1993; Umbriaco et al., 1994). However, in mice, the highest density of M1 immuno-particles is observed in small-caliber oblique dendrites (smaller than 0.66 μm in diameter) of PCs (Yamasaki et al., 2010).

In L5PCs, M2 mAChRs are mainly localized postsynaptically, where they bring about a decrease in excitatory conductances, but M2 and M4 receptors are also present on the cell bodies of GABAergic interneurons in layers 2/3 and 4; here, M2 activation inhibits GABA release. The M3 subtype is localized postsynaptically in rodent inhibitory neurons and dendrites, where it enhances inhibitory transmission (Mrzljak et al., 1993; Groleau et al., 2015). Finally, M4 mAChRs are expressed in cortical excitatory neurons, in particular, in layer 4 spiny stellate neurons (L4SS) across different neocortical regions—S1, V1, and prefrontal cortex (PFC)—where they generate a persistent hyperpolarizing response (Radnikow and Feldmeyer, 2018). Perhaps the presence of M4 mAChRs is a marker to tell apart layer 4 from other layers.

Cholinergic inputs to the cortex generate different responses depending on which receptor is recruited: while M1-like (M1-M3-M5) receptors activation generally leads to an increase in postsynaptic conductance, M2-like receptors (M2-M4) have the opposite tendency to decrease synaptic transmission, by regulating presynaptic ACh release or by directly hyperpolarizing the post-synaptic membrane. mAChRs thus seem to be distributed both at the presynaptic and the postsynaptic level, and the resulting effect depends mostly on which subtype is activated. A detailed understanding of the cellular localization of each receptor subtype for every cell-type is still lacking; some generalizations can be made (as can be seen in Figure 3), but in order to precisely understand how neuromodulatory signals affect neural computation, a detailed knowledge of the amount and distribution of receptor subtypes at the level of each compartment is essential. Furthermore, it is of vital importance to gather this information for each neocortical cell-type. Neuromodulatory inputs very likely affect each cell-type differently, unlocking the possibility of fine-tuning the response and allowing delicate recalibration based on contextual information processing. This is most likely achieved by differentially distributing receptors along cellular compartments, thus creating modulatory micro-domains.

**Figure 1:**
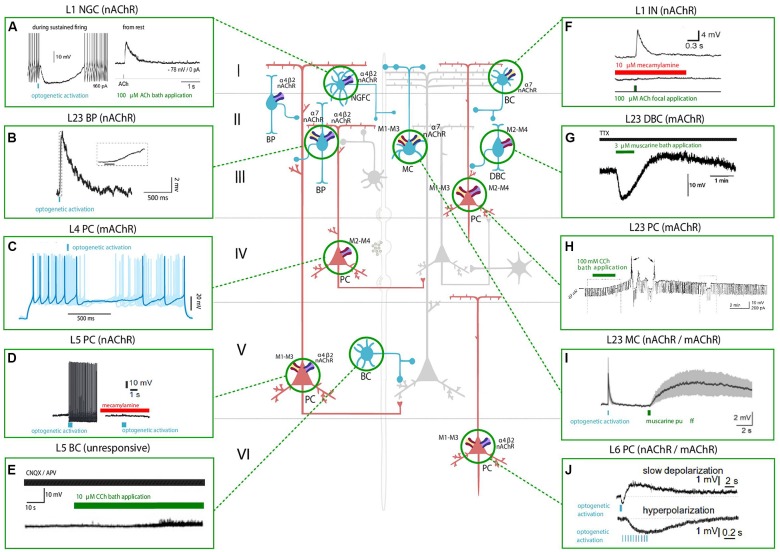
Effect of nicotinic acetylcholine receptors (nAChRs) and muscarinic ACh receptors (mAChRs) activation on the membrane potential of various neocortical cell types. The central schema represents the main cell types in the neocortex. Excitatory neurons are shown in red and inhibitory GABAergic neurons are shown in blue. The electrophysiological responses to the optogenetic activation of cholinergic fibers (in light blue) or the application of a cholinergic agonist (shown in green) or antagonist (shown in red) of each cell type are depicted in the inserts. Timing of cholinergic manipulation is shown as a vertical or horizontal bar. Muscarinic and nicotinic cholinergic receptors associated with the observed response, when known, are shown as four main subtypes: M1-M3-M5 like receptors (yellow and red), M2-M4 like receptors (violet and red), α4β2 heteromeric nAChRs (violet and blue) and α7 homomeric nAChRs (yellow and blue). All shown experimental traces reflect studies listed in Tables s 1, 2. Selected traces were recorded in sensory areas of the rodent neocortex. Inclusion criteria for the experimental traces comprise knowledge of the cell-types and the receptor subtype (nicotinic or muscarinic) involved in the electrophysiological response. Abbreviations: PC, pyramidal cell; SS, spiny-stellate cell; IN, interneuron; MC, Martinotti cell; BC, basket cell; DBC, double-bouquet cell; NGFC, neurogliaform cell; BPC, bipolar cell. Reproduced and adapted from: (left, top to bottom): **(A)**. Brombas et al., 2014; **(B)** Arroyo et al., 2012; **(C)** Dasgupta et al., 2018; **(D)** Hedrick and Waters, 2015; **(E)** Kawaguchi, 1997 (Right, top to bottom): **(F)** Gulledge et al., 2007; **(G)** Kawaguchi, 1997; **(H)** Shalinsky et al., 2002; **(I)** Dasgupta et al., 2018; **(J)** Hedrick and Waters, 2015. For more exhaustive information on agonist concentration, species and cortical area examined, see Tables s 1, 2.

**Figure 2:**
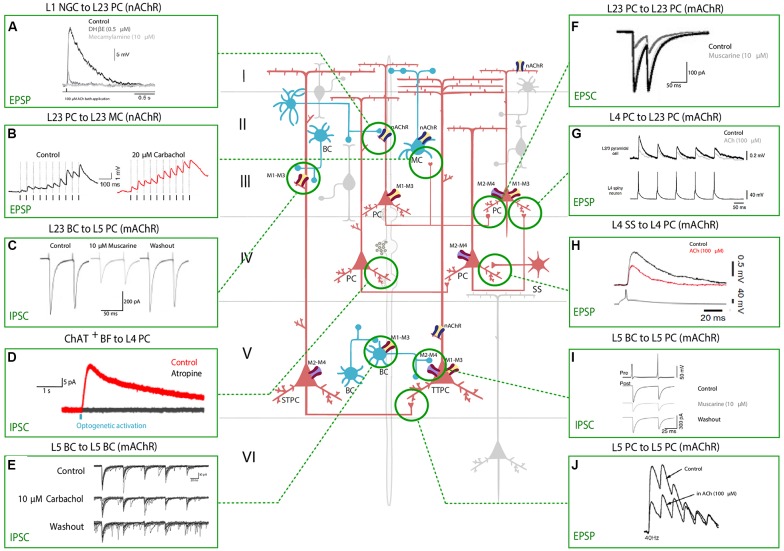
Effect of nAChRs and mAChRs activation on neocortical synaptic dynamics. The central schema represents the main neocortical cell types and their synaptic connections. A fiber of subcortical provenance associated with cholinergic boutons is also shown. Excitatory neurons are shown in red and inhibitory GABAergic neurons are shown in blue. The electrophysiological responses to the application of a cholinergic agonist or antagonist or to basal forebrain (BF) optical stimulation are depicted in the inserts. Panels show the modulation of synaptic dynamics in terms of increase or decrease in PSP/PSC size. Muscarinic and nicotinic cholinergic receptors associated with the observed response, when known, are shown as four main subtypes: M1-M3-M5 like receptors (yellow and red), M2-M4 like receptors (violet and red), α4β2 heteromeric nAChRs (violet and blue) and α7 homomeric nAChRs (yellow and blue). All shown experimental traces reflect studies listed in Table 3. Selected traces were recorded in sensory areas of the rodent neocortex. Inclusion criteria for the experimental traces comprise knowledge of the pre and postsynaptic cell-types and the receptor subtype (nicotinic or muscarinic) involved in the response. Abbreviations: PC, pyramidal cell; TTPC, thick tufted pyramidal cell; STPC, slender tufted pyramidal cell; SS, spiny-stellate cell; MC, Martinotti cell; BC, basket cell; NGFC, neurogliaform cell; BPC, bipolar pyramidal cell; IPC, inverted pyramidal cell. Reproduced and adapted from: (left, top to bottom): **(A)** Brombas et al., 2014; **(B)** Urban-Ciecko et al., 2018; **(C)** Kruglikov and Rudy, 2008; **(D)** Dasgupta et al., 2018; **(E)** Yamamoto et al., 2010; **(F)** Salgado et al., 2007; **(G,H)** Eggermann and Feldmeyer, 2009; **(I)** Kruglikov and Rudy, 2008; **(J)** Markram et al., 1997. For more exhaustive information on technique, species and cortical area examined, see Table 3.

**Figure 3:**
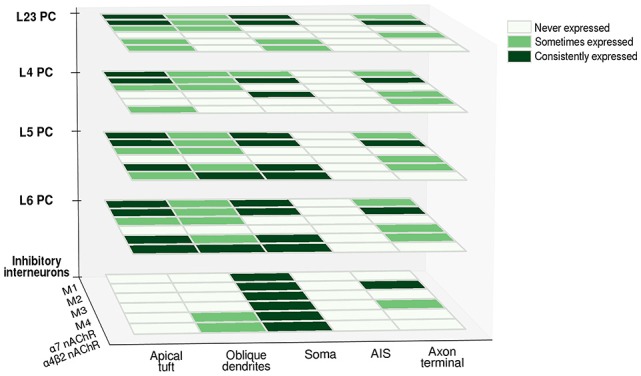
Differential expression of cholinergic receptors in various neuronal compartments across cell-types. Heatmap matrices show the occurrence of cholinergic receptor subtypes at the level of different cell-types. The presence of a given subtype in a cellular compartment is classified as consistently expressed (consistent findings across experimental studies), sometimes expressed (evidence of its presence is only partial) and never expressed (presence of a given subtype is undetectable). Abbreviations: PC, pyramidal cell; M1, M2, M3, M4, muscarinic cholinergic receptors 1–4; nAChR, nicotinic acetylcholine (ACh) receptor.

## Regulation of Neuronal and Synaptic Physiology

ACh can either increase or decrease neurotransmitter release probability, consistent with its role as a neuromodulator rather than a transmitter, and the effect on synaptic release probability depends on the identity of the pre and postsynaptic partners. Cell-types in the neocortex are differentially regulated by ACh, and the effects of cholinergic release include modulation of membrane properties (Figure 1) and synaptic dynamics (Figure 2).

The effects of ACh on neocortical PCs have been thoroughly investigated, and many studies (Gil et al., 1997; Disney et al., 2007) have come to the conclusion that besides generating direct PC depolarization, cholinergic modulation has an overall effect of increasing the signal to noise ratio (SNR) of incoming thalamic inputs. ACh seems to plays a role in enhancing circuit responses to relevant stimuli, providing a mechanism to regulate sensory processing during learning and attention.

The involvement of mAChRs in the depolarizing response of PCs to BF cholinergic inputs has been established by numerous studies (McCormick and Prince, 1985; Delmas and Brown, 2005; Gulledge and Stuart, 2005; Carr and Surmeier, 2007; Zhang and Séguéla, 2010), which report that muscarinic activation in PCs leads to an initial SK-mediated hyperpolarization, followed by a more sustained and slow depolarization (Table 1, Figure 1). Interestingly, the same biphasic response can be induced by bath perfusion of muscarinic agonists in hippocampal interneurons (Heys and Hasselmo, 2012; Heys et al., 2012). The mechanism by which this depolarization emerges has not been fully clarified yet, but some authors suggest the suppression of muscarinic-sensitive and voltage-dependent K^+^ conductance termed the M current (I_m_) or the activation of a non-specific cationic current both support the observed depolarization (McCormick and Prince, 1985; Krnjević, 2004).

**Table 1:** Effect of muscarinic acetylcholine receptors (mAChRs) activation on membrane potential in various neocortical cell types.

Cell type	Receptor	Effect	Area	Technique—Reference
L5 PC	M1 (soma)	Transient hyperpolarization	Rat PMC/V1/PFC	Optogenetics (Hedrick and Waters, 2015)
	M1 (soma)	Slow depolarization	Rat PMC/V1/PFC	1. Optogenetics (Hedrick and Waters, 2015) 2. Somatic puff 100 μM ACh/CCh (Gulledge and Stuart, 2005) 3. 100 μM ACh focally applied (Gulledge et al., 2007)
	M1 (soma)	Hyperpolarization	Rat SSC	100 μM ACh focally applied (Gulledge et al., 2007)
	M1 (soma)	Depolarization	Rat mPFC	30 μm muscarine or oxotremorine bath application (Haj-Dahmane and Andrade, 1996)
L23 PC	Muscarinic	Depolarization	Mouse V1	*In vivo* 2-photon imaging (Alitto and Dan, 2013)
	Muscarinic	Prolonged depolarization	Rat EC layer II	100 mM CCh bath application (Shalinsky et al., 2002)
	M2–M4	Hyperpolarization	Mouse SSC (p12–p16)	Optogenetics (Dasgupta et al., 2018)
L4 PC	M2–M4	Persistent hyperpolarization	Rat SSC	100 μM ACh, puff (Eggermann and Feldmeyer, 2009)
L4 SS	M4 (soma)	Persistent hyperpolarization	Rat SSC	100 μM ACh, puff (Eggermann and Feldmeyer, 2009)
L1 BC	Muscarinic	Depolarization	Mouse V1	*In vivo* 2-photon imaging (Alitto and Dan, 2013)
L1 DBC	Muscarinic	Depolarization	1. Mouse V1 2. Rat PFC	1. *In vivo* 2-photon imaging (Alitto and Dan, 2013) 2. 10 μM CCh or 3 μM muscarine bath application (Kawaguchi, 1997)
L23 DBC	M2	1. Hyperpolarization 2. Hyperpolarization + slow depolarization	1. Rat SSC 2. Rat PFC	1. 100 μM ACh focally applied (Gulledge et al., 2007) 2. 10 μM CCh or 3 μM muscarine bath application (Kawaguchi, 1997)
L23 MC	M1–M3	Depolarization	Mouse SSC	1. Muñoz et al. (2017; M1- M3 KO lines) 2. Optogenetics (Dasgupta et al., 2018)
	Muscarinic	Depolarization	Mouse V1	1 μM/10 mM ACh application (Chen et al., 2015)
L23 BC		Not responsive (NR)	1. Rat SSC 2. Rat PFC	1. 100 μM ACh focally applied (Gulledge et al., 2007) 2. 10 μM CCh or 3 μM muscarine bath application (Kawaguchi, 1997)
L5 BC		NR	Rat SSC	100 μM ACh focally applied (Gulledge et al., 2007)
L5 MC	Muscarinic	NR/slight depolarization	Rat SSC	100 μM ACh focally applied (Gulledge et al., 2007)

*The table links the distribution and localization (when known, in brackets) of muscarinic receptors across neocortical cell types, with respect to cortical layers, with the effect of their activation. The effect of receptor activation is represented in terms of variation of membrane potential. Age of the specimen is given in brackets, when known. When biphasic effects occur, they are listed as multiple effects. Inclusion criteria for the listed studies comprise: (1) recordings performed in the rodent neocortex; (2) knowledge of the morphological type involved; and (3) knowledge of the receptor subtype involved in the response. Abbreviations: PC, pyramidal cell; SS, spiny-stellate cell; IN, interneuron; MC, Martinotti cell; BC, basket cell; DBC, double-bouquet cell; NGFC, neurogliaform cell; BPC, bipolar cell, NBC, nest basket cell; RS, regular spiking. PMC, primary motor cortex; V1, primary visual area; PFC, prefrontal cortex; mPFC, medial prefrontal cortex; EC, entorhinal cortex; SSC, somatosensory cortex, ACh, acetylcholine; CCh, carbachol*.

In L5PCs, transient activation of M1-type mAChRs induces calcium release from IP_3_-sensitive intracellular calcium stores and subsequent activation of an apamin-sensitive, SK-type calcium-activated potassium conductance (Gulledge et al., 2007). Conversely, M4-mediated activation of a potassium conductance (K_ir3_) in L4SS generates a persistent membrane hyperpolarization and induces suppression of neurotransmitter release (Table 1, Figure 1). The observed hyperpolarizing response is supported by a decrease in presynaptic calcium conductance, at synapses between L4PCs and also at synapses between L4PCs and L23PCs (see Table 3, Figure 2; Eggermann and Feldmeyer, 2009). Focal application of ACh onto the soma of L5PCs evokes a biphasic response in which a transient membrane hyperpolarization precedes a slower and longer-lasting depolarization. Pharmacological evidence suggests that this effect is mediated by M1 receptors. Compared with the pressure application of ACh, activation of cholinergic synapses with brief bursts provides relatively weak activation of mAChRs that often fails to affect the somatic membrane potential at rest (Hedrick and Waters, 2015). One possible interpretation of these results might be that synaptically released ACh activates first nAChRs and usually fails to activate mAChRs, whereas pressure ejection onto the soma recruits primarily mAChRs.

**Table 2:** Effect of nAChRs activation on membrane potential in various neocortical cell types.

Cell type	Receptor	Effect	Area	Technique—Reference
L5 PC	α_4_β2 (soma and main dendrite)	Medium depolarization	Mouse PMC/V1/PFC	Optogenetics (Hedrick and Waters, 2015)
	α4 α5	Depolarization Persistent spiking (starting from subthreshold)	Mouse PMC/V1/PFC	Optogenetics (Hedrick and Waters, 2015)
L6 PC	α4 α5 (soma and main dendrite)	Depolarization	Mouse PMC/V1/PFC	Optogenetics (Hedrick and Waters, 2015)
	α_4_β_2_	Depolarization	Rat PFC (p7–p27)	Kassam et al. (2008; bath application of 10 μM ACh to 1 mM)
L1 NGFC	Nicotinic (non-α7)	Depolarization (from RP) Suppression of activity (from subthreshold)	Rat SSC	Iontophoretic application or bath application of 100 μM ACh (Brombas et al., 2014)
L1 BC	Nicotinic	Suppression of activity (at low levels of cortical desynchronization)	Mouse V1	*In vivo* 2-photon imaging (Alitto and Dan, 2013)
L1 INs	Nicotinic	Fast depolarization (from RP)	Rat SSC	100 μM ACh focally applied (Christophe et al., 2002) and (Gulledge et al., 2007)
NBC	Nicotinic	Depolarization	Rat SSC	100 μM ACh focally applied (Gulledge et al., 2007)
BPC	Nicotinic	Depolarization	Rat SSC	100 μM ACh focally applied (Gulledge et al., 2007)
DBC	Nicotinic	Depolarization	Rat PFC	10 μM CCh or 3 μM muscarine bath application (Kawaguchi, 1997)
L23 MC	Nicotinic	Depolarization	Mouse V1	1 μM/10 mM ACh application (Chen et al., 2015)
	Nicotinic	Depolarization	Rat SSC	Optogenetics (Dasgupta et al., 2018)
	α4β2	Depolarization	Mouse S1 and mPFC	Optogenetics or 1 mM ACh bath-application (Obermayer et al., 2018)
L23 BC	Nicotinic	Some are depolarized Some are hyperpolarized	Mouse V1	*In vivo* 2-photon imaging (Alitto and Dan, 2013)
L23 CHAT^+^ BPC	α4β2	Depolarization	Mouse SSC (P20–P40)	Optogenetics (Arroyo et al., 2012)
L23 BPC	α4β2 and α7	Depolarization	Mouse and rat SSC	Optogenetics (Arroyo et al., 2012) and (Dasgupta et al., 2018); 100 μM ACh focally applied (Gulledge et al., 2007)
L5 MC	α4β2	Depolarization	Mouse S1 and mPFC	Optogenetics or 1 mM ACh bath-application (Obermayer et al., 2018)

*The table links the distribution and localization (when known, in brackets) of nicotinic receptors across neocortical cell types, with respect to cortical layers, with the effect of their activation. The effect of receptor activation is represented in terms of variation of membrane potential. Age of the specimen is given in brackets, when known. When biphasic effects occur, they are listed as multiple effects. Inclusion criteria for the listed studies comprise: (1) recordings performed in the rodent neocortex, (2) knowledge of the morphological type involved and (3) knowledge of the receptor subtype involved in the response. Abbreviations: PC, pyramidal cell; SS, spiny-stellate cell; IN, interneuron; MC, Martinotti cell; BC, basket cell; DBC, double-bouquet cell; NGFC, neurogliaform cell; BPC, bipolar cell, NBC, nest basket cell; RS, regular spiking. PMC, primary motor cortex; V1, primary visual area; PFC, prefrontal cortex; SSC, somatosensory cortex, ACh, acetylcholine; CCh, carbachol; RP, resting potential; NR, not responsive*.

**Table 3:** Cholinergic mediated modulation of neocortical synaptic dynamics.

Connection type	Receptor	Effect	Area	Technique—Reference
L5 PC-L5 PC	Muscarinic	Reduction in depression rate of consecutive EPSPs	Rat SSC	Bath application of 50 μM ACh (Tsodyks and Markram, 1997)
	M1 (perisomatic)	Enhancement of EPSCs	Rat SSC (p14–p16)	1–10 μM ACh local puff (Nuñez et al., 2012)
	M2 (basal dendrites)	Reduction of IPSCs	Rat SSC (p14–p16)	50–100 μM ACh local puff (Nuñez et al., 2012)
	Nicotinic	Increase in EPSPs	Rat SSC (p14–p16)	1–10 μM ACh local puff (Nuñez et al., 2012
L5 PC-L5 MC	Nicotinic heteromeric	Decrease in onset delay, increase in time course; no change in EPSP size	Mouse S1 and mPFC	Optogenetics or 1 mM ACh bath-application (Obermayer et al., 2018)
L4 PC-L4 PC	M4	Reduction in first EPSP amplitude	Rat SSC	Bath application of 100 μM ACh (Eggermann and Feldmeyer, 2009)
L4 SS-L4 SS
L4 PC-L23 PC	M4	Reduction in first EPSP amplitude	Rat SSC	Bath application of 100 μM ACh (Eggermann and Feldmeyer, 2009)
L23 PC-L23 PC	M1/M3	Reduction in EPSC amplitude	Rat A1 (p21–p28)	Bath application of 10 μM oxotremorine or muscarine (Atzori et al., 2005)
	Muscarinic (apical dendrite)	Reduction in EPSP amplitude	Rat PFC	Iontophoretic application of 0.05 M muscarine (Vidal and Changeux, 1993)
	Nicotinic (apical dendrite)	Increase in EPSPs	Rat PFC	Iontophoretic application of 0.05 M muscarine (Vidal and Changeux, 1993)
TC fibers-L4 PC	Muscarinic	Increase in EPSP depression rate	Rats and mice TC slice (p21-p28)	Bath application of 5–10 μM muscarine (Gil et al., 1997)
CHAT^+^ fibers—L4 PC	Muscarinic	IPSC	Mouse TC slice (p12–p16)	Optogenetic activation (Dasgupta et al., 2018)
L23 PC-L23 MC	Nicotinic	Increase in EPSPs	Mouse SSC	Bath application of 20 μM CCh (Urban-Ciecko et al., 2018) and optogenetics
	Nicotinic heteromeric	No change in EPSP size	Mouse S1 and mPFC	Optogenetics or 1 mM ACh bath-application (Obermayer et al., 2018)
L1 NGF-L23 PC	M1 (perisomatic)	Connection is silenced (L23 PC is disinhibited)	Rat SSC (p24–p31)	Iontophoretic application or bath application of 100 μM ACh (Brombas et al., 2014)
	Nicotinic (non α7)	Connection is silenced	Rat SSC (p24–p31)	Iontophoretic application or bath application of 100 μM ACh (Brombas et al., 2014)
L23 BC-L5 PC	Muscarinic	Reduction in IPSPs amplitudes (connection is silenced)	Mouse SSC	Bath application of 10 μM muscarine (Kruglikov and Rudy, 2008)
L5 BC-L5 PC	M2/M4	Reduction in u-IPSC amplitude	Mouse SSC	Bath application of 10 μM muscarine (Kruglikov and Rudy, 2008)
	Muscarinic	Reduction in u-IPSC amplitude	Rat insular cortex	Bath application of 10 μM CCh (Yamamoto et al., 2010)
L5 MC-L5 PC	Nicotinic heteromeric	Decrease in onset delay, no change in IPSP size	Mouse S1 and mPFC	Optogenetics or 1 mM ACh bath-application (Obermayer et al., 2018)
	Nicotinic	Increase in IPSP size	Mouse A1	Bath application of 10 μM CCh (Hilscher et al., 2017)
L5 BC-L5 BC	Muscarinic	Decrease in IPSCs amplitudes	Rat insular cortex	Bath application of 10 μM CCh (Yamamoto et al., 2010)
L5 RS IN-L5 PC	Muscarinic	Decrease in IPSCs amplitudes	Rat insular cortex	Bath application of 10 μM CCh (Yamamoto et al., 2010)
L5 BC-L5 RS IN	Muscarinic	Increase in first IPSCs amplitudes	Rat insular cortex	Bath application of 10 μM CCh (Yamamoto et al., 2010)
L5 RS IN-L5 RS IN	Muscarinic	Increase in first IPSCs amplitudes	Rat insular cortex	Bath application of 10 μM CCh (Yamamoto et al., 2010)

*The table links the distribution and localization (when known, in brackets) of nicotinic and muscarinic receptors across neocortical cell types, with respect to cortical layers, with the effect of their activation on synaptic dynamics. Effect is represented in terms of increase or decrease in PSP/PSC size. Age of the specimen is given in brackets, when known. Inclusion criteria for the listed studies comprise: (1) recordings performed in the rodent neocortex; (2) knowledge of the pre and post synaptic morphological types involved; and (3) knowledge of the receptor subtype involved. Abbreviations: PC, pyramidal cell; SS, spiny-stellate cell; IN, interneuron; MC, Martinotti cell; BC, basket cell; DBC, double-bouquet cell; NGFC, neurogliaform cell; BPC, bipolar cell, NBC, nest basket cell; RS, regular spiking. A1, primary auditory area; PFC, prefrontal cortex; SSC, somatosensory cortex; TC thalamo-cortical; ACh, acetylcholine; CCh, carbachol; EPSP, excitatory post-synaptic potential; IPSP, inhibitory post-synaptic potential; EPSC, excitatory post-synaptic current; IPSC, inhibitory post-synaptic current*.

Muscarinic activation modulates K^+^ conductances (McCormick, 1992), but the reversal potential for K^+^ is approximately −90 mV: mAChR activation, therefore, exerts a little effect at resting membrane potential. However, when a neuron is depolarized, the observable mAChR-mediated hyperpolarization and subsequent depolarization are larger. The reported biphasic effect affects both cortico-pontine (CPn) and commissural (COM) pyramidal neurons; however, COM neurons show a more pronounced inhibitory phase, while CPn neurons have a larger and longer-lasting depolarizing phase (Baker et al., 2018). While these effects have been characterized thoroughly in deep-layers PCs, others report that ACh has limited ability to inhibit superficial PCs *via* changes in membrane potential (Gulledge et al., 2007).

Cortical inhibitory interneurons are, as well as PCs, a prominent target of cholinergic neuromodulation. The ways in which ACh modulates the dynamics of local interneurons have not been completely clarified yet, because the effects of BF cholinergic stimulation and bath application of cholinergic agonists (Table 1) strongly depend on the inhibitory cell-type.

Exogenous application is unlikely to mimic accurately the spatiotemporal profile of ACh release from cholinergic axons, and furthermore, there seems to be no agreement within the neuroscientific community on which concentration of cholinergic agonists should be used to promote activation of the cholinergic receptors. The applied dose ranges from 10 to 100 micromolar across different experimental groups, and in other cases, it even spans the millimolar range. These discrepancies arise from the fact that to measure the physiological extracellular concentration of ACh is experimentally challenging, because of the prompt intervention of hydrolases in the synaptic cleft. Application of acetylcholinesterase inhibitors cannot be avoided, making it extremely difficult to detect physiological levels of ACh in the extracellular space. Moreover, while mAChR agonists have been extensively used and are known to generate a multitude of responses in cortical neurons, much fewer studies (Hedrick and Waters, 2015; Dasgupta et al., 2018) have discerned muscarinic responses evoked by endogenous ACh release (see Figures 1, 2).

Cholecystokinin-immunoreactive (CCK) cells are affected heterogeneously by cholinergic agonists depending on their sizes. For example, small CCK cells are promptly depolarized by cholinergic inputs, while bigger CCK cells show a biphasic response comprising an initial hyperpolarization and a subsequent depolarization similarly to PCs (Kawaguchi, 1997). There is a general consensus (Gulledge et al., 2007; Kruglikov and Rudy, 2008; Poorthuis et al., 2013) that cholinergic modulation of fast-spiking PV positive (PV^+^) interneurons does not produce any effect on membrane excitability (Table 1). However, evidence also shows the opposite. For example, Alitto and Dan (2013) report in their review that PV^+^ interneurons are depolarized *via* muscarinic activation, but when mAChRs are blocked by antagonist application, the excitation is converted to inhibition; in turn inhibition of PV^+^ cells is converted to excitation when nAChRs are blocked, suggesting that excitation and inhibition compete in the same population of PV^+^ interneurons through the activity of the different receptors.

The subpopulation of dendrite-targeting interneurons, that is identified as somatostatin (Sst) expressing (Sst^+^) interneurons (MCs), can be depolarized by activation of mAChRs (Fanselow et al., 2008). However, some studies report that only very few Sst^+^ interneurons display excitation or inhibition in response to BF stimulation and that the inhibitory cells displaying the strongest excitation by ACh are L1 and VIP^+^ interneurons). Recent findings outlined by Muñoz et al. (2017) challenge these results. In their study, they claim that cholinergic modulation of Sst^+^ interneurons *via* M1 and/or M3 mAChRs provides a major excitatory drive to these cells during whisking activity.

VIP expressing interneurons are highly responsive to cholinergic inputs and show a mixed activation profile that is partially blocked by both nicotinic and muscarinic receptor antagonists (Kawaguchi and Kubota, 1997).

In summary, muscarinic activation has differential effects on membrane potential, based on which subtypes are expressed in a specific cell-type and in cellular compartments. These heterogeneous responses might play different roles in neocortical information processing: the initial hyperpolarizing phase observed in PCs and some CCK^+^ cells could be used to push the cell away from threshold, while the subsequent depolarization selectively augments inputs that are strong enough to reach threshold, therefore increasing the SNR, and at the same time promoting synchronization of neural activity. At the same time, the presynaptic inhibition of excitatory feedback could serve as a mechanism to prevent interference during the encoding of new stimuli and reduce top-down influences on perceptive processes. In addition, muscarinic receptors contribute to the generation of the gamma rhythm by inducing synchronized oscillations in both excitatory and inhibitory neurons (Heys et al., 2012).

Another class of receptors contributes to cholinergic signaling in the neocortex. Nicotinic receptors exert fast cortical actions, playing a key role in many cognitive processes (Dani and Bertrand, 2007), as described in the following section (Dani and Bertrand, 2007).

## Nicotinic Receptors

ACh is primarily regarded as a neuromodulator rather than a neurotransmitter in the CNS because its physiological effects have a latency of onset of tens of milliseconds to minutes (Van der Zee and Luiten, 1999). This great variability in the response of cortical neurons to ACh stimulation originates from the fact that there are two main types of ACh receptor proteins. Neuronal nicotinic receptors (nAChRs) are ionotropic receptors which are composed of combinations of twelve different nAChR subunits: α2 to α10 and β2, β3, β4. Each receptor is made of five subunits. It is generally assumed that nicotinic actions are fast and precise; however, the depolarization rate produced by the opening of the nicotinic channel can vary depending on the specific subunit composition. Because mAChR signaling acts through G-proteins, mAChR signaling might be expected to be slower than ionic nAChR signaling. However, homomeric (α7) nAChRs can also mediate slow responses, and the time course of muscarinic action may also vary widely, depending on the signal pathways involved (Muñoz and Rudy, 2014).

The nicotinic branch of the AChR family can be further divided into at least two classes, based on the affinity that their binding sites have for nicotine itself or the snake toxin α-bungarotoxin. At their simplest neuronal nAChRs are homomeric (constituted from five identical subunits) while the more complex forms are heteromeric, composed of at least one α and one β subtype. Binding studies using [^3^H]-nicotine have shown that high-affinity nAChR binding sites are very common for the human cerebral cortex, while autoradiographic labeling of nAChRs shows an inhomogeneous distribution over architectonically identified cortical areas of the rat brain, with highest concentrations in the medial PFC (mPFC) and generally frontal areas.

As for mAChRs, the expression of different subunit combinations varies across layers and across cortical areas. Given the involvement of the nicotinergic system in the treatment of tobacco addiction, many studies have been performed in the human brain. Most data on the distribution of nAChRs has been obtained from human autopsy tissue homogenates using techniques such as ligand binding, RT-PCR, immunoprecipitation, and Western blot.

Currently-available nAChR agonists and antagonists used for receptor auto-radiography are not subtype specific, although they act on nAChR subtypes with a distinct profile: labeling experiments carried out with different probes revealed that nAChRs are widely expressed in the cortex, both at the level of gray and white matter; many fibers show immunoreactivity at the neuropil level (Schröder, 1992). Five α subunits (3–7) and three β subunits (2–4) are expressed in the human brain. The expression of α4 and β2 subunits in the frontal cortex, parietal cortex, and temporal cortex shows a characteristic laminar distribution. Higher receptor binding is observed in layers 1, 3 and 5. These results are in agreement with the observed distribution of α3 and α4 mRNAs that are mostly found in PCs of layer 2/3 and layer 5 of the frontal cortex (Wevers, 2011). However, other studies report that the α3 mRNA is exclusively expressed in layer 4, while α4 subunit is moderately expressed in all layers (Radnikow and Feldmeyer, 2018). The α7 subunit is found mostly in layer 1–3 and 5 and is virtually absent in layer 4, while α4 and β2 immunoreactive fibers were observed in layer 4 of the PFC (Sparks et al., 2018). The α2 subunit is a characteristic feature of L5MCs that project to layer 1 and specifically target L5TTPCs (Hilscher et al., 2017). The detection of nicotinic subunits is possible because of the existence of specific antisubunit-antibodies and the introduction of nAChR subunit-Cre mouse lines. Nevertheless, nicotinic receptors are made up of multiple subunits and are either homomeric or heteromeric. The most abundant receptor subtypes in the neocortex are the homomeric receptor α7 and the heteromeric α4β2 channel (which is often associated with the regulatory subunit α5; Radnikow and Feldmeyer, 2018). Nicotinic receptors can be activated both *via* volume transmission and fast synaptic activity (Dani and Bertrand, 2007; Hedrick and Waters, 2015; Hay et al., 2016).

## Pre-synaptic Localization

None of the studies mentioned above investigates the precise cellular localization of cholinergic receptors, which is crucial in determining the outcome of the response. This is especially true for nAChRs, because their activation directly leads to a cation influx into the cell, and immediately results in a voltage change in the underlying compartment.

nAChRs are expressed on glutamatergic inputs to layer 5, mostly contacting layer 5 interneurons and L5/L6 PCs. L5PCs and L6PCs are modulated by α7 and β2 nAChRs, respectively, while L23PCs and glutamatergic inputs to these cells do not contain nAChRs. Interneurons across layers contain mixed combinations of nAChRs (Poorthuis et al., 2013). Some subtypes, such as α7 homomeric receptors, are preponderantly expressed in presynaptic areas, whereas heteromeric receptors are more expressed on cell bodies and main dendrites (Bertrand, 2010). Cholinergic axons that diffusely innervate the cortex are thought to make *en passant* connections in the area of the main dendrite of the PCs from layer 5 and VI, therefore causing a volume release of ACh. Pre-synaptically, nAChRs generally increase the release of GABA and glutamate (Dani and Bertrand, 2007). However, both nAChR and mAChRs can reduce EPSPs by acting pre-synaptically (Levy et al., 2006).

## Post-synaptic Localization

The distribution of nAChRs at the light and electron microscopic level was studied in the human cerebral cortex using anti-nAChR monoclonal antibody (mAb) WF-6, which is not subunit selective (Schröder et al., 1990): nAChR immunoreactivity revealed a pattern for the frontal and temporal cortex that was very similar to that obtained with the auto-radiography. In the frontal cortex, *in situ* hybridization techniques display numerous labeled neurons, mostly PCs bearing the α7 mRNA in the cell body and in the apical dendrite. In the motor cortex, many PCs showed signals in the proximal part of their apical dendrite.

As reported by Schröder et al. (1989) and Schröder (1992) nAChR localization is predominant in L23 and L5 PCs; a few nAChR-expressing fusiform cells can be detected in layer 4 and VI. Many PCs show nAChRs on basal dendrites that originate in layer 5, cross the superficial layers of the cortex perpendicular to the pial surface, and branch between layers 1 and 2. Immuno-precipitate is detectable both in cell bodies and in their apical dendrites, in branches of various diameters, and in the PSD of synaptic junctions. In a double-labeling approach conducted in the temporal cortex, it was further demonstrated that PV^+^ interneurons express α4 and α7 subunit protein (Wevers, 2011). Double-labeling studies have shown that at least 30% of cortical neurons contain both nAChR and mAChR proteins, the majority of these being PCs. In the human cortex, nicotinic immuno-staining in individual neurons appears generally comparable to that seen in the rodent model (Schröder et al., 1989; Schröder, 1992): as in the rat occipital cortex, nAChRs can be detected on the cell bodies and dendrites of L23 and L5 PCs.

Most studies agree that nAChRs are preferentially found in infragranular layers, mostly at the level of L5 and L6PCs, but also at the level of inhibitory interneurons; CB-immunoreactive neurons, as well as PV^+^ neurons all express nAChRs, while that is not true for CR-ir neurons (Coppola and Disney, 2018); furthermore, nAChRs are expressed at the level of layer 2/3 as well, both in PC bodies and in the apical dendrites of deeper-layer placed cells. However, only a small subset of layer 2/3 excitatory neurons and no layer 4 neurons express nAChRs; layer 6 expression profile can be set apart from the rest, given that these neurons predominantly express the slowly desensitizing heteromeric α4β2 channel (Radnikow and Feldmeyer, 2018).

The distribution of nAChRs and the subunits combination, therefore, depends on cell-types, laminar position and on the cortical area studied, similarly to mAChRs; nowadays the possibility of systematically studying the distribution profile of cholinergic receptors has greatly increased, due to the advancement in the production of anti-subunit-specific-antisera and to the development of better immunoprecipitation and ligand binding techniques. Such studies exist and are quite informative as regards, for instance, the striatum (Zoli et al., 2002), but a comprehensive and detailed investigation of the expression of subunits in the neocortex is still lacking. Nicotinic activation prevalently modulates the excitability of deep cortical layers: in the next section, we move on and explore the contribution of nicotinic stimulation to local circuit properties and examine studies that investigated the involvement of the nicotinergic system in the modulation of neocortical activity.

## Regulation of Neuronal and Synaptic Physiology

Even though nAChRs are predominantly expressed pre-synaptically, where their activation modulates neurotransmitter release through calcium influx or terminal depolarization (Nashmi and Lester, 2006), there is evidence that nAChRs may also influence post-synaptic signaling and that these effects vary based on the subcellular localization of the receptor (Tables s 2, 3). nAChRs expressed on distal dendrites are thought to cause the generation of fast excitatory post-synaptic potentials since activation of nAChRs on distal apical dendrites promotes PC depolarization and leads to an increase in action potential firing. On the contrary, activation of nAChRs on the proximal apical dendrites (closer to the cell body) reduces membrane impedance and shunts signal incoming from the apical tuft: when the nAChRs opens, the membrane resistance of the PC decreases and signals incoming from the apical dendrites get attenuated (Dani and Bertrand, 2007).

Optogenetic activation of cortical cholinergic input generates an increase in membrane excitability (Table 2) mediated by nAChRs and promotes spiking in L5PCs (Hedrick and Waters, 2015). When the stimulation is paired with additional depolarization, spiking activity becomes persistent and can be blocked by BAPTA application, suggesting that the observed depolarization is mediated by intracellular Ca^++^ transients. As suggested by kinetic analysis it is likely that non-α7 nAChRs determine this response. The depolarizing response spans all layers, but occurs with laminar and regional differences; additionally, the effect of the depolarization can be moderate and transitory or pronounced and persistent depending on the cell membrane potential. Although the modulatory effect was found to be stronger in deeper layers, the authors report that it was similar in M1, V1 and prefrontal (PF) cortices. The preferential modulation of deep neocortical layers is likely to influence the flow of excitation occurring throughout the neocortex that originates in layer 4 and then propagates to the superficial layers, whose role is to modify the output of layer 5. Altogether this study showed that nAChR activation increases the excitability of neocortical PCs; in the light of previous evidence that α4 and α5 subunits are highly expressed in layer 6 (Tribollet et al., 2004), and nAChR-mediated responses in layer 6 of the PFC have already been reported by many studies (Kassam et al., 2008; Bailey et al., 2010; Poorthuis et al., 2013), the authors suggest that the presence of α4 and α5-mediated PSPs could be a characteristic feature of L6PCs across neocortical regions (see Table 2, Figure 1).

Pyramidal-to-PCs connections in layer 5 can be potentiated by using an spike-timing-dependent-plasticity (STDP) protocol. Bath-application of 10 μM (or 300 nM) nicotine impairs L5PC to L5PC potentiation and favors the induction of LTD. When monitoring spontaneous synaptic events, application of nicotine increases the frequency and amplitude of sEPSCs. Evoked excitatory post-synaptic currents (EPSCs) behave differently and are reduced in amplitude by nicotine. However, puffing nicotine directly on PCs fails to elicit an inward current, and application of gabazine prevents the de-potentiation. Therefore, the effects of nicotine on L5PC to L5PC synapses should be attributed to an enhancement of GABAergic transmission, rather than the direct activation of a PCs (Couey et al., 2007).

nAChRs are known to be distributed throughout the dendritic trees of cortical PCs (van der Zee et al., 1992), but a comprehensive mapping of cholinergic synapses apposition remains elusive. To provide concomitant information on receptor localization while recording electrical responses more researchers should apply the strategy used by Hedrick and Waters (2015), who measured nicotinic PSPs during restricted illumination of the slice: illumination of the tuft dendrites failed to evoke a nicotinic PSP at the soma and therefore the authors concluded that nAChRs that contribute to the somatic depolarization are likely to be within 300 μm of the soma and many are probably located in the proximal 50 μm of the apical and basal arbor. This technique sheds light on the compartmental origin of the observed response and it is immensely useful to causally link the distribution of cholinergic receptors and their physiological role. A subsequent investigation should combine this strategy with pharmacological inactivation of specific receptor subunits and provide further proof that PCs responses to cholinergic inputs in different layers are mediated by specific receptor subunits and that their distribution profile is greatly involved in determining the outcome of neural computations.

Although nAChRs are mainly found on PCs, there is extensive evidence that nAChRs are expressed on the membrane of cortical interneurons (Table 2), such as MC, chandelier cells (ChCs) and basket cells (BCs), where they contribute to the modulation of GABAergic signaling (Couey et al., 2007; Wevers, 2011). The subpopulation of serotonin receptor 5-HT3aR expressing GABAergic interneurons is depolarized by ACh *via* nAChRs (Gulledge et al., 2007; Poorthuis et al., 2013); this embryologically distinguished subpopulation, that accounts for about 30% of the total number of cortical inhibitory interneurons, is heterogeneous and includes all the VIP^+^ interneurons, as well as the VIP^−^ neurogliaform cells (NGCs; Rudy et al., 2011). VIP^+^ interneurons show a mixed activation profile in which both nicotinic and muscarinic receptors are involved (Figure 1; Kawaguchi, 1997).

Prominent nAChRs expression is a hallmark of layer 1 inhibitory interneurons both in rodents and humans (Letzkus et al., 2011; Alitto and Dan, 2013) and endogenous cholinergic release is known to rapidly recruit this receptor subpopulation during locomotion and attentive processes. These fast, nicotinic responses are mediated by α7 and β2 containing receptors (Poorthuis et al., 2018). When at rest, all layer 1 interneurons are depolarized *via* nicotinic activation (Figure 1, Table 2); however, when these interneurons are engaged in repetitive firing, ACh inhibits the activity of L1 NGCs (Brombas et al., 2014). Conversely, single bouquet cells (SBCs) are activated by ACh in the regime of repetitive firing (Jiang et al., 2013). Layer 1 interneurons responses are abolished by application of nAChR antagonists (Figure 1; Christophe et al., 2002).

ACh enhances the activation of neocortical deep-layers PCs by ascending thalamic inputs *via* mAChR-mediated depolarization and subsequent enhanced glutamate release from thalamocortical terminals in layer 4 (Gil et al., 1997; Metherate and Hsieh, 2004; Disney et al., 2007), but it also releases inhibition on superficial layers PCs. There is extensive evidence that ACh mediates activation of layer 1 and layer 2/3 non-fast spiking PV^−^ cortical interneurons *via* non-α7 nAChRs. These interneurons, in turn, inhibit MCs and BCs that directly target PCs: nAChR-mediated inhibition of superficial interneurons reduces inhibition of superficial PCs (Gulledge et al., 2007; Arroyo et al., 2012; Brombas et al., 2014). Photostimulation of ChAT^+^ neurons in the BF evokes a prolonged disynaptic inhibition in PCs; pharmacological manipulation of the response suggests that it is supported by non-α7 mediated excitation of specific interneurons subtypes. This finding indicates that nicotinic cholinergic input originating from BF fibers is also comprised of a slow component. The observed delayed barrage of inhibitory post-synaptic current (IPSC) in L23PCs exhibits a long latency (of about 26 ms) characteristic of dysynaptic inhibition. Layer 1 and layer 2/3 inhibitory interneurons, and in particular in late-spiking cells and L23 ChAT^+^ bipolar cells are responsible for this phenomenon (Arroyo et al., 2012). In agreement with previous reports (Poorthuis et al., 2014) fast-spiking cells such as BCs and ChCs do not exhibit EPSPs in response to optogenetic stimulation of ChAT^+^ BF neurons, but rather respond similarly to PCs and are swamped by an IPSC barrage as well. While layer 1 and layer 2/3 late spiking cells (LS) exhibit both a fast and a slow response, L23 ChAT bipolar cells display only a slow response. This study demonstrates that the fast and slow components are mediated by α7 receptors and non-α7 receptors, respectively, and that non-α7 receptor-mediated excitation elicits action potentials in cortical interneurons that in turn produce a delayed and prolonged wave of inhibition in L23PCs and FS cells. One proposed explanation for the slow response is that it may arise from a cholinergic bulk transmission and that it may sustain the high metabolic demand of processes such as attention and memory (Cauli et al., 2004). Cortical ChAT^+^/VIP^+^ interneurons have been shown to dilate local microvasculature to increase blood supply during periods of elevated neuronal activity (Kocharyan et al., 2008) during the execution of memory and attention tasks, following electrical BF stimulation. The fast component of the cholinergic response may also be implicated in the emergence of a broader phenomenon like synchronized neuronal activity; it has been shown that LS cells are connected *via* gap junctions, and this fast response may thus play a fundamental role in the emergence of network oscillations that sustain plasticity and attention mechanisms.

Couey et al. (2007) realized that the effect of nicotine on L5PC to L5PC connections is mostly due to an enhancement of GABAergic transmission, and they decided to dissect the effects of nicotine on three different interneurons types. First, they looked at the activity of FS cells in layer 5, and observed no effect when adding nicotine to the bath; later they stained the cells for certain neuropeptides and several nAChR subunits and found an extremely low amount of mRNA coding for nicotinic subunits in FS cells, which might explain their unresponsiveness. Once again, another piece of evidence emerges confirming that (putative) BCs have a tendency not to respond to the application of cholinergic agonists. The authors identified another type of interneuron as a regular-spiking-non-PC (RSNPC), and observed a fast inward current after application of nicotine. LTS cells (putative MC) showed an even bigger inward current response; in both cell-types the most abundantly stained nicotinic subunit was α4, but β2 and α7 were also present. In this study, nicotine application increases the frequency and amplitude of spontaneous EPSCs in putative BCs and MCs; as for putative ChC (RSNP) a decrease in the frequency, but not the amplitude of sEPSCs can be observed (Couey et al., 2007).

Pyramidal to SST^+^ interneurons neocortical connections are relatively weak, but local excitatory input to SST neurons is selectively enhanced during cholinergic modulation of network activity. In a recent 2018 study, it was shown that endogenous ACh release activates presynaptic nAChRs and boosts glutamatergic input in a target-cell specific manner (Urban-Ciecko et al., 2018). Thus, there is evidence that local excitatory input to SST neurons is selectively enhanced during nicotinic modulation of network activity (Table 2, Figure 2). In a recent study by Obermayer et al. (2018) examined PC-MC-PC disynaptic connections in both layer 2/3 and layer 5 and found that the typical delayed disynaptic inhibitory response in the post-synaptic PC is faster and stronger when cholinergic inputs are activated optogenetically, or by means of 1 mM ACh bath application. When looking at the activity of a single MC, they observed that ACh inputs lead to a significant decrease of the onset delay of AP firing and increases the number of APs fired in MCs, which can account for the earlier onset and prolonged duration of disynaptic inhibition. This effect was abolished by application of 10 μM DHβE demonstrating that it is mediated by heteromeric nicotinic receptors (Table 2, Figure 2). However, when they recorded from synaptically connected PC-MC pairs during concurrent activation of cholinergic fibers, they could only observe an increase in the membrane depolarization level, but not in EPSP sizes. The same effect was found in MC-PC connections, and this as well was confirmed to be nicotinic in nature, contradicting the result obtained by Urban-Ciecko and others and others. The setups of the two experiments are comparable: both studies were performed in the adult mouse somatosensory cortex. However, the first remarkable difference lies in the nature of the cholinergic input used in the two experiments: while Obermayer et al. (2018) used bath-application of 1 mM ACh and optogenetic activation, Urban-Ciecko et al. (2018) decided to record activity in the presence of 20 μM CCh, a non-hydrolyzable analogue of ACh. Not only the two concentrations differ by two orders of magnitude, but the two cholinergic agonists work in fundamentally different ways. While ACh is almost immediately hydrolyzed by the cholinesterase in the synaptic cleft (within a few milliseconds), carbachol has a much more prolonged effect (Katz and Miledi, 1973). Nevertheless, the results obtained by bath-application of ACh are in agreement with the results achieved by optogenetic activation of the cholinergic system, which is supposed to be a more physiological way of stimulating cholinergic release (Obermayer et al., 2018).

Interestingly, optogenetic activation of cholinergic inputs did not affect the typical fast disynaptic post-PC response mediated by BCs, which provides yet another example of how BCs tend to be unresponsive to cholinergic release in both layer 2/3 and layer 5, or more generally show a more heterogeneous response profile to ACh inputs (Obermayer et al., 2018). This could be explained by the lack of a precise morphological identification of various subtypes of BCs, which could express cholinergic receptors in different subcellular locations or in a different amount, and therefore show differential responses to ACh inputs. These findings indicate that subcortical neuromodulatory projections recruit nicotinic receptors to alter network function through increased inhibition and provide a potential mechanism by which attention controls the gain of local circuits.

## Nicotinic and Muscarinic Kinetics

What are the receptor affinities to various agonists and can this be related to the actual amount of nicotinergic modulation? The relative activation of receptors vs. the concentration of agonist has been measured (Table 4).

**Table 4:** Nicotinic homomeric and heteromeric receptors kinetics.

Receptor type	Single channel conductance	Open time	PO_max_	EC50 for ACh or nicotine	Kinetics
Nicotinic heteromeric (α3)2(β4)3	29 pS	0.71 ± 0.14 and 3.5 ± 0.4 ms			
Nicotinic heteromeric (α3)2(β4)3	29 pS (Stetzer et al., 1996), 18.2 ± 0.46 (Rovira et al., 1998)	147 ms (Stetzer et al., 1996)			
(α3β2)2α5				EC50 ACh 1.70–1.83 μM for ACh EC50 Nicotine 2.91 μM IC50 Nicotine 2.92 μM (Kuryatov et al., 2011)	Fast: 40–121 ms; slow: 274–1039 ms (Figl and Cohen, 2000)
(α3β4)2 α5				EC50 Ach 115–122 μM EC50 Nicotine 4.64 IC50 Nicotine 16.7 μM (Kuryatov et al., 2011)	
Nicotinic heteromeric (α4)2(β2)3	31.3 pS, 40.5 pS (high state) and 21.9 pS (low; Hales et al., 2006)	207 ± 38 ms (Hsiao et al., 2008)	0.8 (Li and Steinbach, 2010)	High affinity is 1.6 μM, low affinity is 62 μM (Buisson and Bertrand, 2001) EC50 for ACh in activating α7 is 3 μM (Albuquerque et al., 2000)	Fast 4–6 ms; slow 30–53 ms (Figl and Cohen, 2000)
Nicotinic heteromeric (α4β2)2 α5				EC50 ACh 1.44–1.64 μM for variants tested EC50 Nicotine 0.62 μM IC50 Nicotine 0.0872 μM (Kuryatov et al., 2011)	
Nicotinic homomeric (α7)5	82.9 pS (Albuquerque et al., 2000)	108 μs and 92.7 μs for channels activated by 11 and 10 mM Ach, respectively (Albuquerque et al., 2000)		Choline: EC50 1.6 mM; IC50 37 μM (Alkondon and Albuquerque, 2004). 200 μM ACh (Buisson and Bertrand, 2001). EC50 for ACh in activating α7 130 μM (Albuquerque et al., 2000)	

*The table lists properties of nicotinic homomeric and heteromeric receptors (single-channel conductance, open time and open probability and EC50 and kinetics)*.

Muscarine reversibly reduces Ca^2+^ currents in a dose-dependent manner. The modulation is rapid, with an onset time constant of 1.2 s. A slowly developing component of the modulation also is observed, with a time constant of 17 s. Under elevated Ca^2+^ conditions, the fast component is due to a reduction in both N- and P-type calcium currents, whereas the slow component involves L-type current (Stewart et al., 1999). Receptor properties such as conductance, open time, and sensitivity to ACh depend on the nicotinic subunit composition (Table 4). (α4)2(β2)3 nAChRs are sensitive to micromolar scale changes, while (α7)5 receptors have a half-maximal sensitivity of more than a hundred micromolar. Extracellular choline is normally 3–5 μM but can attain 20 μM in some pathological cases. However, ACh reaches the millimolar range at the site of release (Alkondon and Albuquerque, 2004). Responses mediated by α_7_ nAChRs are short-lasting, whereas those mediated by α_4_β_2_ nAChRs are long-lasting. This is because the mean open time of α_7_ nAChRs is shorter than that of α_4_β_2_ nAChRs. Also, α_7_ nAChRs desensitize much faster than α_4_β_2_ nAChRs (Alkondon et al., 1999).

An interesting hypothesis was put forward by Albuquerque et al. (2000). α_7_ but not α_4_β_2_ nAChRs can be fully activated by choline (Nguyen et al., 1995; Alkondon et al., 1999). Choline and acetate are the products of hydrolyzation of synaptically released ACh by ACh-esterase in the synaptic cleft. This process occurs quickly, but reuptake of choline into presynaptic terminals is slow. Therefore, the ACh concentration in the synaptic cleft should decay rapidly, with only low levels of diffusing ACh reaching peri-synaptic sites. But choline levels should rapidly rise in the synaptic cleft with high levels of diffusing choline reaching peri-synaptic sites. This implies that extrasynaptically located α4β2 nAChRs (i.e., the high affinity nAChRs) could be activated by diffusing, low levels of ACh, extrasynaptically located while low-affinity α7 nAChRs may be activated by diffusing choline. Thus, α7 and α4β2 nAChRs might exhibit differential control (Albuquerque et al., 2000).

## Subcellular Nicotinic and Muscarinic Pathways

**Figure 4:**
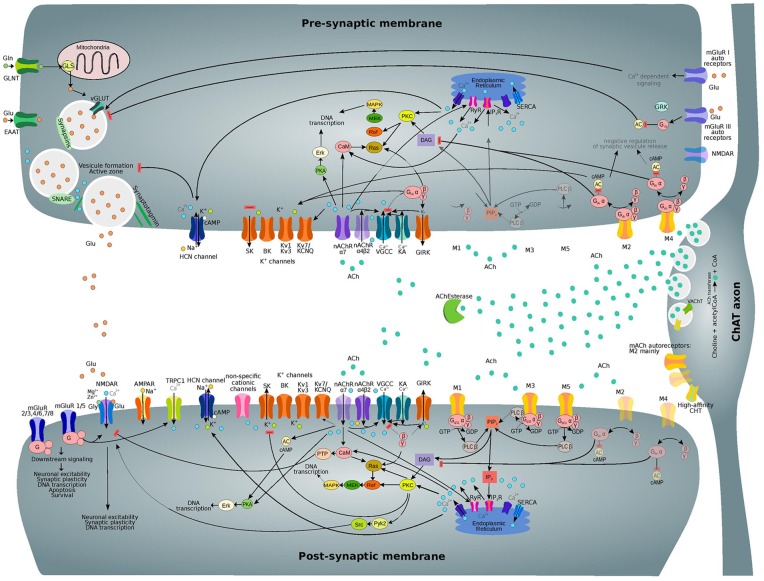
Subcellular nicotinic and muscarinic signaling processes at the glutamatergic synapse being modulated by ACh. Only the main relevant pathways and components are shown. Receptor subtypes which are less expressed on pre and post-synaptic membranes and related downstream processes are shown in semi-transparent colors. Abbreviations: ACh, acetylcholine; ACh Esterase, acetylcholinesterase; M1-M5, muscarinic acetylcholine receptor types 1–5; nAChR (α7, α4β2), nicotinic acetylcholine receptor (types α7, α4β2); VGCC, voltage-gated calcium channel; KA, kainate receptor; GIRK, G-protein activated inward rectifier K^+^ channel; PKA, protein kinase A; CaM, calmodulin; AC, adenylyl cyclase; DAG, diacylglycerol; PKC, protein kinase C; NOS, NO-synthase; HO-2, heme oxygenase 2; sGC, soluble guanylyl cyclase; PKG, cGMP-dependent protein kinase; HCN, hyperpolarization-activated cyclic nucleotide-gated channel; TRPC1, transient receptor potential cation channel 1; mGluR, metabotropic glutamate receptor; Pyk2, protein-tyrosine kinase 2; PiP2, phosphoinositol-1,4,5-biphosphate; PLC β, phospholipase C β; IP_3_, inositol triphosphate; IP_3_R, IP_3_ receptor; RyR, ryanodine receptor; SERCA, sarco-endoplasmic reticulum Ca^2+^-ATPase.

ACh affects membrane conductance through several subcellular pathways, as illustrated in Figure 4, leading to both hyperpolarizing and depolarizing effects (Tables s 1, 2). ACh can act on both pre and post-synaptic membranes, binding to muscarinic and nicotinic receptors. The interplay among intracellular pathways leads to a dynamically changing outcome, such as the transient hyperpolarization and following long-term depolarization resulting from the binding of ACh to M1 mAChR (Dasari et al., 2017). When ACh interacts with M1, the exchange of coupled GDP for GTP produces the dissociation of the G-protein complex from the receptor. The released α subunit of the Gq protein then activates the enzyme phospholipase C (PLC β) which hydrolyzes phosphatidyl-inositol 4,5 bisphosphate (PIP_2_), leading to its dissociation from the membrane and the subsequent formation of diacylglycerol (DAG) and IP_3_. IP_3_ initiates calcium ions release from the endoplasmic reticulum (ER), serving as a trigger for this process. Refilling of the ER with Ca^2+^ions is then obtained by the activity of the sarco-ER Ca^2+^-ATPase (SERCA). Extracellular calcium ions are therefore crucial for the maintenance of calcium cycling. M1 activation facilitates voltage-dependent refilling of calcium stores by promoting excitation. Thus, fine-tuned calcium dynamics govern complex reciprocal relations among many different proteins contributing to changes in membrane potential. Ultimately, changes in K^+^, Ca^2+^-activated K^+^-currents and non-specific cationic currents support a shift from transient hyperpolarization to a sustained excitation.

Meanwhile, DAG together with Ca^2+^ ions activate kinases such as protein kinase C (PKC), causing multiple downstream effects. PKC controls the function of many proteins including members of both pre and post-synaptic membranes. PKC is also involved in synaptic plasticity regulation and causes the internalization of AMPARs and NMDARs, leading to LTD phenomena (Callender and Newton, 2017).

PKC can also phosphorylate metabotropic glutamate receptor 5 (mGluR5; Hwang et al., 2005) as well as many other proteins. Moreover, PKC activates heme-oxygenase 2 (HO-2; Artinian et al., 2001) and inhibits NO-synthase (NOS), interfering with the calcium/calmodulin activation of NOS enzyme (Borda et al., 1998). These effects contribute to the downstream processes involving carbon monoxide (CO) and nitric oxide (NO) as interacting messengers (Mathes and Thompson, 1996; Artinian et al., 2001). Long-term effects of PKC activation include changes in DNA transcription that are mediated by MAPK/Erk signaling. Furthermore, there is recent evidence for the direct interaction of M3 mAChR with PLC β, which increases signaling efficiency (Kan et al., 2014).

The downstream signaling pathways of M3 and M5 receptors overlap with that of M1, and therefore they are grouped as M1-like receptors; similarly, M2-type mAChRs comprise both M2 and M4 receptors. Binding of ACh to M2-type mAChRs results in the inhibition of adenylyl cyclase (AC) by the α subunit of G_i/o_ protein and in the subsequent reduction of cAMP levels (Muñoz and Rudy, 2014). However, there are some differences between the G_i_ and G_o_ mechanisms of AC regulation (Jiang and Bajpayee, 2009). The βγ-complex of the dissociated G-protein can activate the G-protein activated inward rectifier K^+^ channels (GIRK) and inhibit voltage-gated calcium channels (VGCCs). Moreover, G_o_ proteins can also regulate Na^+^ channels (Jiang and Bajpayee, 2009). Particular effects of M1 and M2 receptors on different ion channels have been already summarized by Thiele et al. (2012).

A significant increase in intracellular calcium concentration comes from the direct flow of ions due to the permeability of nAChRs to Ca^2+^. However, nAChR activation also leads to the activation of VGCC and subsequent Ca^2+^ influx. (Dajas-Bailador and Wonnacott, 2004; Shen and Yakel, 2009). Moreover, functional cross-talk among presynaptic nAChRs has been shown to affect signal transduction (Marchi and Grilli, 2010). Therefore, the action of one receptor might depend on the function of co-existing receptor subtypes in the same cell. The interaction between presynaptic nicotinic receptors with other ionotropic or metabotropic receptors serves the purpose of producing an integrated response.

## Transcriptome Cell-Specific Prediction of Cholinergic Receptors

In recent years, a wealth of transcriptomic data from the mouse brain has become available (Saunders et al., 2018; Zeisel et al., 2018). Many different cell types may exist; one study found 565 different cell groups, for example (Saunders et al., 2018). Since a standard classification of cortical cell types is still emerging, most articles employ different approaches to arrive at cell type specific transcriptomes.

**Figure 5:**
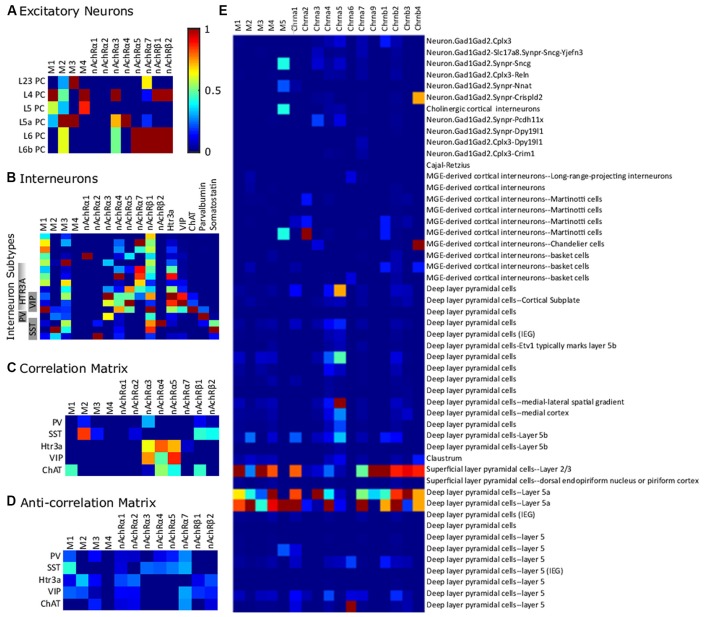
Differential expression of cholinergic receptors in transcriptome-derived cell types. **(A)** Excitatory cell types. **(B)** Interneurons in somatosensory cortex. Gene expression is normalized to a maximum of 1 on a gene-by-gene basis. **(C)** Correlation matrix (positive values of correlation matrix Pearson correlation coefficient matrix). **(D)** Anti-correlation matrix (negative values of correlation matrix). The data is from Zeisel et al. (2018) and was collected with high-throughput single-cell RNA sequencing, a method which counts individual RNA molecules. Abbreviations: PV, parvalbumin; SST, somatostatin; VIP, vasointestinal peptide; ChAT, choline acetyltransferase. **(E)** Expression of ACh receptor genes across the Frontal cortex cell-clusters identified in Saunders et al. (2018). The data was collected using Drop-seq (a method which allows the use of older animals and elimination of certain technical artifacts) to profile the RNA expression of individual cells. Semi-supervised independent component analysis was used to group cells into the sub-clusters using network-based clustering (ibid). Expression levels were normalized to the highest expression across all the selected genes. In this data set, receptor expression was particularly high in L23 and L5a PCs.

We examined a representative data set from the somatosensory cortex in order to interpret possible cell-specific differences in cholinergic receptor expression (Figure 5). We chose this data set since excitatory cell types are mapped to layer-specific types, allowing the easiest comparison with the types referenced in this review. In this dataset, normalized expression of M1 receptors is highest in L4 PCs. There is a strong expression of M2 in deep layer neurons, particularly in layer 5a. M3 is highly expressed in layer 2/3 and layer 5a, while M4 is highest in layer 4. α3 nAChR subunits are highest in layer 4, but also in the deep layers. β subunit expression is highest in layer 6 and layer 6a neurons. Inhibitory interneuron expression of cholinergic receptors is definitely cell-type specific, though heterologous. PV cells express more nAchRα3 than do somatostatin-expressing interneurons (Figure 5B). Somatostatin expression is best correlated with M2 expression and nicotinic β subunit expression and negatively correlated with M1 expression (Figure 5C). VIP and Htr3a expression is correlated with nAchRα3, nAchRα4, and nAchRα5. Furthermore, ChAT expression is correlated with M1 expression. In layer 5a, the effects of the predominantly-expressed nAChR and mAChRs seemed to be synergistic.

We also examined an additional dataset for frontal cortex (Figure 5E; Saunders et al., 2018). M5 is expressed in a subset of interneurons, including some cholinergic and MCs. The nicotinic receptor Chrna5 is expressed in a subset of deep PCs. Chrna6 is most expressed in a particular type of layer 5 PC. This dataset illustrates that the degree of sub-classification of PCs is likely to be important. For example, there are many subtypes of L5PCs, which have different cholinergic receptor expression. Both datasets showed consistency in M3 expression in L2/3 and L5a PCs but not L4 and L5 PCs.

In addition to cell-type specific correlation, nAChR genes that encode heteromeric α/β subunits are well correlated among themselves (Zoli et al., 2015; Saunders et al., 2018). The genes encoding the α subunits correlate well with the corresponding β subunit.

Cholinergic neurons can be identified by cluster analysis (Zeisel et al., 2018). In particular, separate types have been identified in the red nucleus and habenular nucleus of the thalamus (ibid). ACh often is released in neurons releasing other neurotransmitters (Zeisel et al., 2018). In the habenular nucleus, the glutamate transporter Slc17a6, in cholinergic cells, suggesting co-release of glutamate and ACh (Mancarci et al., 2017). In the ventral midbrain, a neuron type that was both dopaminergic and cholinergic was identified (Zeisel et al., 2018). Many forebrain cholinergic neurons also are GABAergic (Mancarci et al., 2017), consistent with the co-release of these two substances (Saunders et al., 2015).

## Global Network Effect and Modulation of Brain States

The transition between different brain states that occurs whenever an organism switches from one behavioral state to another is associated with changes in the overall pattern of neural activity, which can be captured with EEG or LFP recordings. The pattern of EEG activity can change dramatically with the behavioral state of the animal (Lee and Dan, 2012), as can be seen in the transition from slow-wave sleep to wakefulness (or from deep sleep to REM sleep), when the EEG pattern shifts from large and synchronous waves of neural activity to a more desynchronized and short-amplitude wave pattern (Berger, 1929). Ensemble neuronal activity undergoes impressive changes during behavioral state transitions, and different brain states have been associated with different brain functions; definitive evidence for these functions although, is still lacking, and the mechanism by which these transitions are achieved in the cortical network is not yet understood. Many authors have proposed that the switch between cortical states may be driven by the action of neuromodulators like ACh (Lee and Dan, 2012). However, precisely how these neuromodulators influence global cortical processing by locally targeting specific cells is largely an unsolved mystery.

## Basal Forebrain Modulation of Brain States

A large body of evidence suggests that the BF, a complex and heterogeneous structure classically defined by the presence of clusters of cholinergic neurons, is crucial for the maintenance of the sleep/wake cycle and for processes that underlie arousal and attentional modulation, but it is unclear which BF neurons promote each brain state and how they interact with each other to regulate transitions between states (Anaclet et al., 2015). Already since 1930, it was known that BF lesions could cause severe insomnia (Saper et al., 2001); however, this evidence has been an object of constant challenge over the years, and the attempts to replicate this experiment would yield different results. Finally, Szymusiak and McGinty (1986) observed that sleep-active cells were confined to the ventral BF in the cat (the horizontal limb of the diagonal bands of Broca, substantia innominata, entopeduncular nucleus and ventral globus pallidus) and that these areas partially overlap with those where chemical and electrical stimulations evoke sleep, and where lesions suppress sleep. The sleep-active cells were thus considered optimal candidates for mediating some of the sleep-promoting functions attributed to the BF (Szymusiak and McGinty, 1986).

Many BF neurons are active during wake and during REM sleep (Lee and Dan, 2012), and specific lesions reduce wakefulness, in agreement with the finding that BF lesions cause significant increases in delta waves occurrence during wakefulness, and that BF stimulation induces cortical desynchronization of EEG or LFP signals, accompanied by a decrease in correlated spiking. Furthermore, the BF receives inputs from the LDT and PPT pontine nuclei; cholinergic neurons that can be found at the level of the LDT nucleus exhibit an increase in firing rate during cortical activation, just before the transition from slow-wave sleep frequencies to faster frequencies (Saper et al., 2010).

Therefore, it seems reasonable to hypothesize the existence of functionally diverse neurons in the BF: according to Duque et al. (2000), BF cells that exhibit different wake/sleep activity pattern, also express different molecular markers (Zaborszky and Duque, 2000). There are three major neuronal types in the BF: cholinergic, glutamatergic and GABAergic cells (Anaclet et al., 2015; Xu et al., 2015). There might be extensive local synaptic interactions among BF neurons mediating local reciprocal inhibition between GABAergic neurons and sleep-active and wake-active cholinergic neurons. The well-known flip-flop circuit for sleep/wake cycle control (Saper et al., 2010) could, therefore, comprise multiple loops and switches. However, some findings suggest that BF GABAergic neurons provide major contributions to wakefulness, while cholinergic and glutamatergic neurons appear to play a lesser role; chemogenetic activation of GABAergic neurons promotes wake and high-frequency EEG activity, whereas cholinergic or glutamatergic activation have a destabilizing effect on slow-wave-sleep (SWS), but has no effect on total wake (Anaclet et al., 2015).

Cholinergic neurons residing in the BF can be divided into two subpopulations, that might be involved in different functions: an early-spiking population may reflect phasic changes in cortical ACh release associated with attention, while the late-spiking group could be more suited for the maintenance of the cholinergic tone during general cortical arousal (Unal et al., 2012).

## Multi-Transmitter Neurons: ACh and GABA Co-transmission

Nevertheless, functional co-transmission of ACh and GABA seems to be a common feature of nearly all forebrain ACh-producing neurons (Henny and Jones, 2008; Granger et al., 2016). BF inputs to the neocortex are therefore not only constituted of different fibers, but also use a mixture of functionally diverse neurotransmitters (Kalmbach et al., 2012). This opens the question of whether there is a substantial difference between the cholinergic modulation and the BF modulation of neocortical activity. The contribution of GABA needs to be considered when studying the functional impact of ACh-producing neurons: electrical stimulation of BF fibers might evoke markedly different responses than optogenetically-evoked selective cholinergic release.

Does the co-release happen in a target-specific modality, at different terminals branching from the same axon, or is the release site the same for both transmitters? And if so, how does GABA affect the ongoing cholinergic modulation? Release of an excitatory (ACh) and inhibitory (GABA) neurotransmitter by the same axons seems to be functionally antagonistic. However, both transmitters could act in parallel, depending on the mode of co-transmission (Granger et al., 2016). If both ACh and GABA are released simultaneously onto the same post-synaptic cells, then GABA may act to shunt the (supposed) excitation generated by ACh. Otherwise, they could target different postsynaptic cells, such that GABA inhibits one cell population while ACh excites another. Given previous experimental results showing that GABA release from VIP interneurons shunts activity of Sst^+^ interneurons, but not other VIP interneurons, it is thought that VIP/ChAT cortical interneurons may release ACh and GABA onto different post-synaptic targets, perhaps from separate synaptic vesicle populations (Granger et al., 2016). Indeed, a recent analysis of the molecular composition of the pre-synaptic terminals of cortical VIP/ChAT interneurons revealed that ACh and GABA vesicles are confined to separate boutons. At the post-synaptic level, the subset of GABAergic boutons seems to contact prevalently other inhibitory interneurons, while ACh boutons target mostly L1 interneurons and other VIP/ChAT cortical interneurons. Here, ACh evokes EPSCs that are mediated by nicotinic receptors (Granger et al., 2018). Another recent study conducted in the mPFC confirms that only 10%–20% of post-synaptic targets of VIP/ChAT cortical interneurons are contacted by both cholinergic and GABAergic inputs (Obermayer et al., 2018); here they report that VIP/ChAT neurons directly excite interneurons in layers 1–3 as well as PCs in L2/3 and L6 by fast nicotinic transmission.

Immunolabeling studies (Beaulieu and Somogyi, 1991) have shown substantial co-labeling of presynaptic cholinergic terminals for both GABA and ChAT in the neocortex, but more studies should address the functional consequences of the synaptic co-release of these neurotransmitters and try to dissect the differential impact of each transmitter on postsynaptic cells excitability. Analysing the co-localization of post-synaptic receptors or scaffolding proteins could also allow the identification of individual synapses that are sensitive to both ACh and GABA. These possibilities should be addressed systematically in order to precisely understand the contribution of each neurotransmitter to cortical processing.

## ACh Involvement in Neuroplasticity

Apart from the fine-tuning of sleep/wake transitions, cholinergic neuromodulation is tightly implicated in regulating selective attention to a given sensory stimulus by altering the activity of the sensory cortex that perceives that modality (Kim et al., 2016). ACh is known to be especially involved in cortical arousal (Saper et al., 2010) and in the state-dependent modulation of cortical activity; cholinergic neurons are active during locomotion (Buzsaki et al., 1988) and during transition to the attentive state (Kim et al., 2016). Studies have shown that the occurrence of relevant sensory events evokes a transient increase in ACh concentration in the rat PFC (Hasselmo and Sarter, 2011). Conversely, activating cholinergic transmission in the PFC determines an improvement in subject’s performance during sustained attention tasks (Saper et al., 2010). It is, therefore, reasonable to hypothesize that ACh can induce long-lasting changes in neuronal excitability, and indeed this was demonstrated. Pioneering experiments showing that ablation of noradrenergic and cholinergic innervation in the striate cortex substantially impairs ocular dominance plasticity in kittens (Bear and Singer, 1986) opened the way for subsequent studies on the involvement of ACh in cortical plasticity. Some showed that when a tone is paired with NBM stimulation or ACh application, auditory cortex receptive fields change and prolonged enhanced responses to the paired frequency can be observed (Metherate and Weinberger, 1990; Rasmusson, 2000). Others discovered that co-application of muscarinic agonists with glutamate induces a prolonged increase in response to glutamate in somatosensory cortical neurons (Sugihara et al., 2016), and that these effects concern as well the somatosensory cortex and the primary visual area V1. According to Metherate and Weinberger (1990), the potentiation can be blocked by cortical application of atropine, but others (Sugihara et al., 2016) report that cholinergic antagonists cannot reverse the prolonged changes, thereby confirming that ACh is necessary for the induction, but not the maintenance of these modifications. ACh seems to act more as an instructive, rather than a permissive signal (Lin et al., 2015).

ACh is as well involved in the generation of LTD at synapses between cortical pyramidal neurons and striatal medium spiny neurons through disinhibition of Ca_v_ channels. Here, the activation of D_2_ receptors reduces basal ACh release from cholinergic striatal interneurons and lowers M_1_ receptor tone in medium spiny neurons, which leads to enhanced opening of intraspine Ca_v_1.3 Ca^2+^ channels in response to synaptic depolarization. The calcium transient results in enhanced production of endocannabinoids (ECs) such as 2-arachidonoylglycerol, and activation of presynaptic CB_1_ receptors that reduce glutamate release (Wang et al., 2006).

Furthermore, the role of several neuromodulatory systems in STDP induction (Pawlak et al., 2010) has been studied across multiple brain areas. While dopamine (DA) and NA modulation of STDP has been mostly investigated in subcortical areas, ACh’s role in STDP induction has been extensively researched in neocortical sensory areas and in the PFC. In mouse mPFC, nicotine application increases the threshold for STDP in L5PCs by reducing their dendritic calcium signals. This effect, however, is due to an enhancement in GABAergic transmission in various types of interneurons in the PFC network, that express multiple types of nAChRs (Couey et al., 2007), and not to a direct nicotinic action on PCs.

Taken together, evidence suggests that cholinergic inputs to the cortex incoming from the BF should be viewed more as teaching, rather than motivational signals. Overall, activation of the cholinergic system controls the shift from a correlated or synchronized state, to a decorrelated or desynchronized state and results in an enhancement of cortical information processing (Lee and Dan, 2012). However, exactly how the detection of relevant stimuli is enhanced and which are the mechanisms at the basis of this ACh-induced desynchronization are still a matter of open debate.

## ACh Enhancement of Sensory Processing

NBM stimulation has a differential effect on spontaneous and sensory-evoked activity. In a recent study, Meir et al. (2018) showed that NBM stimulation desynchronizes cortical LFP and increases the SNR of sensory-evoked responses while suppressing ongoing spontaneous synaptic activity. The authors recorded spontaneous PSPs occurring in L4 and showed that following NBM stimulation the frequency and amplitude of sPSPs were decreased. Moreover, the mean membrane voltage of the response became more hyperpolarized, and trial-to-trial variability was decreased, both during spontaneous and evoked activity. However, sensory stimulation did not change the amplitude of the response, whereas it caused a prominent reduction in the noise amplitude, therefore changing the SNR of the sensory response. By analyzing the coupling of Vm and LFP signals, they also showed that cholinergic activation largely reduced fluctuations in the membrane potential and caused a decorrelation in network activity.

Chen et al. (2015) were able to identify a defined microcircuit in the superficial layers of mouse V1 that supports ACh driven desynchronization. The authors measured the activity of different inhibitory interneurons while optogenetically stimulating superficial cholinergic axons, and found that cholinergic inputs facilitate Sst^+^ interneurons, which in turn inhibit PV^+^ interneurons and PCs. Optogenetic inhibition of Sst^+^ neurons blocks desynchronization, whereas direct activation of Sst^+^ neurons is sufficient to induce desynchronization (Chen et al., 2015). The observed desynchronization in cortical activity may explain the role of ACh in mediating transitions between phases of the sleep-wake cycle, but it fails to explain how ACh enhances sensory processing. A large body of evidence suggests that ACh enhances sensory inputs while simultaneously suppressing intrinsic cortical activation (Kimura et al., 1999; Disney et al., 2007; Newman et al., 2012), but a detailed understanding of this process is currently lacking. ACh’s role may substantially differ across sensory areas and affect different tuning properties.

Nucleus basalis activation affects sensory responses to natural stimuli of a population of cortical neurons. Before BF stimulation, multi-unit activity (MUA) in the rat’s V1 is highly correlated but poorly time-locked to the stimulus; after BF stimulation it becomes less correlated but more time-locked to the sensory event. NBM stimulation also decreases single-unit activity (SU) correlation (between cells correlation) and increases response reliability (between trials correlation coefficient) but does not induce any significant change in receptive field size, orientation tuning nor direction selectivity. Atropine application decreases NBM induced decorrelation, indicating that mAChRs support this effect (Goard and Dan, 2009). After NBM stimulation a shift in the firing modality of the LGN resembling that found at the level of the thalamus can be observed, namely a transition from burst to tonic mode (Bazhenov et al., 2002; Castro-Alamancos and Gulati, 2014). A similar study (Thiele et al., 2012) was conducted in the extrastriate cortex of the macaque and yielded opposing results: at the level of the middle temporal (MT) area it revealed how other tuning properties, like orientation and direction discriminability, are also affected by cholinergic modulation; in this case, ACh had little effect on response reliability, though it is still not clear whether these differences are attributable to differences existing between rodents and primates or to functional differences between sensory areas. In an effort to clarify the precise role of neocortical cholinergic modulation, Disney et al. (2007) concentrated on the role of nAChRs in a well-studied cortical model system, the V1 of the macaque monkey. Here they showed *in vivo* that nicotine reliably enhances the gain of responses to visual stimuli in layer 4c, but not in other layers. Having found β2-nAChR in a pre-synaptic position at the level of thalamo-cortical synapses on PV^+^ interneurons, they prove that nicotine enhances detection of visual stimuli through enhanced TC transmission. These findings confirm that cholinergic activation causes an increase in cortical sensory responses through enhancement of thalamic synaptic transmission and suppression of intracortical inputs. A systematic effort to extend these results to other sensory areas is therefore needed in order to decipher whether the mechanism supporting cholinergic modulation is common throughout all cortical areas or if different tuning properties are affected each time.

## ACh Modulation of Thalamo-Cortical Transmission

Castro-Alamanco and Gulati recorded, multi-electrode activity (MUA) and field potential from adult rat barrel cortex following multi-whisker stimulation at 0.2 Hz, while increasing concentrations of carbachol or other drugs were applied by means of micro-dialysis. The authors found that the application of 50 μM carbachol, but not norepinephrine, can stop the emergence of the 10–15 Hz oscillations that are observed during baseline recordings and that in the presence of atropine these oscillations are even enhanced (Castro-Alamancos and Gulati, 2014). The effect of carbachol on barrel cortex LFP is thus congruent with the traditionally termed desynchronization for doses higher than 50 μM (Moruzzi and Magoun, 1949; Steriade et al., 1993). A low tone of cholinergic activation (0.5–1 μM) however, reinforces the deactivated cortical state by enhancing synchronous slow oscillations. A very high tone of cholinergic activation (250–2,500 μM) leads to a significant increase in tonic firing, without altering the overall firing rate. An interesting follow-up to this experiment would be to check whether the same effect can be observed in the whole somatosensory region, and across other sensory cortices. The group then tried to decipher whether cholinergic activation would also modulate thalamocortical activity: by recording from the VPM, they found that cholinergic cortical activation suppresses burst-firing in the thalamus and changes neuronal firing to a tonic mode. This result is fairly consistent with the outcome predicted by the model of thalamo-cortical slow-wave sleep oscillations and transition to activated states generated by Bazhenov et al. (2002). Here, the increase in ACh activity was modeled by the reduction of a K^+^ leak current in pyramidal and thalamo-cortical cells and resulted in the abolishment of the hyperpolarizing phase of network activity and a consequent increase in the input/resistance relationship, accompanied by a switch to the tonic firing (15–20 Hz) modality. The transition from bursting to tonic firing thus seems to be a characteristic feature of relay diencephalic structures like the thalamus and the meta-thalamus.

Enhanced thalamo-cortical transmission seems to be a constant finding across a vast number of articles and reviews (Bazhenov et al., 2002; Disney et al., 2007; Hasselmo and Sarter, 2011) with the aim of revealing the mechanisms by which cholinergic neuromodulation operates. Next studies in this field should, therefore, consider the possibility that cholinergic inputs reach the cortex not only through direct BF projections but also exploiting the thalamo-cortical loop.

Voltage-sensitive dye imaging revealed that ACh application to the neocortex, upon stimulation of layer 2/3, suppresses the spread of excitation to nearby areas. Thus, ACh seems to play an important role in coding sensory stimuli by enhancing thalamocortical inputs, but at the same time, by suppressing intracortical interactions (Kimura et al., 1999).

One of the proposed models for the cholinergic mediated shift from default mode to detection mode suggests that ACh acts to enhance the glutamatergic representation of thalamic input through stimulation of nAChRs, while suppressing the cortical spread of associational input through activation of mAChRs (Hasselmo and Sarter, 2011). Minces et al. (2017) recently evaluated the effect of increases in cortical ACh following optogenetic BF stimulation on the correlation structure of the visual network and found that transient cholinergic release in the cortex decreases the slope between signal and noise correlations. The authors propose that this mechanism acts to increase the encoding capacity of the network.

Another article evaluated the impact of ACh on local circuit activation and found that cholinergic inputs exclude unreliable neurons from contributing to circuit activity while conserving neurons that were active in response to thalamic activity and showed strong correlations. Moreover, weak functional connections were pruned, thus yielding a more modular and hierarchical circuit structure. Once again, these results highlight how ACh is able to reorganize the circuit function in a way that promotes the discriminability of thalamic inputs at the expense of weak pairwise relationships (Runfeldt et al., 2014).

## Sensory Modality-Specific Information Processing and ACh

Many studies (Disney et al., 2007; Minces et al., 2017) have focused on trying to understand the role played by ACh in improving stimuli detection or modifying receptor fields size in the visual cortex. While many of them have been done in primates, others have privileged the somatosensory areas and highlight the involvement of the cholinergic system in the regulation of sensory cortical processing in rodents as well, supporting the idea that cholinergic modulation of cortical microcircuits is functionally equivalent across brain areas and model organisms, even though a canonical and anatomically equivalent system is not strictly identifiable (Coppola and Disney, 2018).

The finding that distinct neuronal clusters in the BF project selectively to specific sensory areas (Kim et al., 2016) and that cholinergic inputs to sensory cortices are spatially segregated supports the idea that cholinergic release improves sensory discrimination in a modality-selective manner and with a high degree of specificity. The authors mapped BF projections to different sensory areas and found retrobead-labeled neurons from three different sensory cortices within the BF, with a clear distinction between the clusters of cells: neurons in the HDB project preferentially to V1, the posterior part of NBM projects to A1, while the aNBM preferentially projects to S1. These results were further confirmed by another experiment in which the authors optogenetically activated cholinergic neurons in the BF subnuclei and successfully induced modality-selective desynchronization in specific sensory cortices.

A similar experiment was performed by Chaves-Coira et al. (2016), who also used retrograde anatomical procedures to demonstrate the existence of specific neuronal groups in the BF implicated in the modulation of specific sensory cortices. However, here the authors found that most of the neurons located in the HDB projected to the S1 cortex, suggesting that this area is specialized in the sensory processing of tactile stimuli, and the NBM was found to have a similar number of cells projecting to S1 as to A1. Furthermore, optogenetic HDB stimulation induced a larger facilitation of tactile evoked potentials in S1 than auditory evoked potentials in A1, while optogenetic stimulation of the NBM facilitated either tactile or auditory evoked potentials equally. These results suggest that cholinergic projections to the cortex are organized into spatially segregated pools of neurons that modulate specific cortical areas; although, additional research will be needed in order to provide a clear and definitive picture of the topographical organization of the projections arising from the BF region and innervating the cortex. Despite the many attempts to clarify this issue, it remains unclear whether there exist distinct neuronal populations in the HDB, or whether the differences observed in the outcomes of the experiments mentioned above are due to discrepancies existing in the transgenic mouse lines used or to the slightly different techniques that were employed.

ACh is thus involved both in the bottom-up attentional process that leads to a general and whole-state arousal of the cortex and in the top-down modifications of circuit activity that occur during detection of behaviorally relevant sensory stimuli. Cognitive functions of cholinergic projection systems vary according to the brain area that is being modulated. Cholinergic modulation may act as a common mechanism to improve sensory encoding in several brain areas.

## Summary and Outlook

ACh release in the neocortex controls transitions between brain states, such as attention, memory and wakefulness, and can occur through volume or synaptic transmission. However, it is not clear yet whether one modality prevails upon the other or if they are complementary mechanisms. Further studies are needed to establish correlations between the distribution profile of the receptor subtypes, the relative proximity and density of cholinergic varicosities to assess differences between the two modalities. Moreover, as results could vastly vary across species, a systematic effort is crucial to be able to compare quantitative measurements.

The expression of muscarinic and nicotinic cholinergic receptors—the two main types—varies according to the cell-type and the pattern of receptor localization varies across cortical layers. A detailed knowledge of the subcellular localization of cholinergic receptors is, however, currently lacking. The detection of cholinoceptive structures such as the receptor protein has become easier with the advent of polyclonal antibodies targeting different subtypes. Future investigations should, therefore, converge on systematically measuring the amount of each receptor subtype across cellular compartments.

In this review, we have endeavored to determine, in a quantitative manner, the cellular and synaptic effects of ACh release in the neocortex. While the cholinergic modulation of excitatory PCs has been extensively researched, its effect on inhibitory interneurons is still largely unknown. For example, the effect of ACh on BCs (fast-spiking, PV^+^ interneurons) remains unclear. This could be due to the lack of a thorough classification of diverse morphological types of BCs where a differential distribution of cholinergic receptors could modulate divergent cellular and synaptic effects. Furthermore, it is not clear whether bath-application of cholinergic agonists is comparable to a physiological activation of the cholinergic system. Applied concentrations of cholinergic agonists vary substantially (up to three orders of magnitude) across electrophysiological studies, which seldom use more than one concentration. To obtain carefully designed dose-response curves of the effects of cholinergic agonists is paramount to dissect the consequences of physiological ACh release in the neocortex. The advent of optogenetics holds promise in designing physiological protocols of ACh release. Future experiments should not only merely employ traditional bath-application of cholinergic agonists but also exploit optogenetics to reconcile how doses of agonists directly map to effects of endogenous, physiological release of ACh.

The effects of ACh on synaptic connections can vary drastically according to the identity of the presynaptic terminal and its postsynaptic partner. Additionally, the magnitude of the postsynaptic response also depends on the receptor subtype being activated. Therefore, there is a clear requirement for systematic investigations of the effects of ACh on different synapse-types, combined with knowledge of implicated cell-types and receptor subtypes to unravel the effects of ACh release on necortical synaptic transmission.

ACh is involved in the induction of synaptic plasticity mechanisms, which could support its role in cortical learning and memory. In addition, ACh enhances sensory processing by affecting receptor fields size and tuning properties. It is not clear, however, if the effects of ACh are modality-specific or can be generalized to all sensory processing, nor exactly which tuning properties are affected. Many studies point to a role of ACh in increasing the SNR of a sensory response, and others describe how ACh suppresses cortico-cortical interactions in favor of thalamic transmission. Therefore, further clarification is required on the matter. Moreover, special attention must be paid in integrating data from primates and rodents: neuromodulatory systems are commonly the object of evolutionary modifications, even though they might maintain some functional similarity throughout species.

The mechanisms of ACh-induced changes in the physiology of neocortical neurons and their synapses, and how these changes shape the emergence of global network states still remains elusive. The impact of ACh on global cortical computations sustains cognitive functions such as attention, learning and memory, which are characterized by desynchronized network activity. Cholinergic inputs mainly originate in the BF, a structure comprising distinct multi-transmitter neuronal populations. The functional relevance of neuronal subpopulations in the BF and the co-release of two potentially antagonistic transmitters to the desynchronization of cortical activity is unknown. Furthermore, recent work identifies that a sub-population of VIP^+^ cortical interneurons co-release ACh and GABA with potentially differing functions across species. Future research should, therefore, focus on dissecting the impact of each transmitter on cellular excitability. In addition, analyzing the co-localization of post-synaptic receptors could also allow the identification of individual synapses that are sensitive to multiple neurotransmitters. All these possibilities should be addressed systematically in order to precisely understand the contribution of each neurotransmitter to ACh-induced effects on the emergence of cortical network states in health and disease.

## Author Contributions

CC, DK, PS and SR wrote the manuscript and drafted the figures and tables. SR, DK and HM reviewed and edited the manuscript and the figures. SR conceived the idea and supervised the study.

## Conflict of Interest Statement

The authors declare that the research was conducted in the absence of any commercial or financial relationships that could be construed as a potential conflict of interest.
